# Large-Scale Coarse-to-Fine Object Retrieval Ontology and Deep Local Multitask Learning

**DOI:** 10.1155/2019/1483294

**Published:** 2019-07-18

**Authors:** Ngoc Q. Ly, Tuong K. Do, Binh X. Nguyen

**Affiliations:** ^1^Department of Information Technology, VNUHCM-University of Science, HCM 70000, Vietnam; ^2^Researcher at AIOZ Pte Ltd, HCM 70000, Vietnam

## Abstract

Object retrieval plays an increasingly important role in video surveillance, digital marketing, e-commerce, etc. It is facing challenges such as large-scale datasets, imbalanced data, viewpoint, cluster background, and fine-grained details (attributes). This paper has proposed a model to integrate object ontology, a local multitask deep neural network (local MDNN), and an imbalanced data solver to take advantages and overcome the shortcomings of deep learning network models to improve the performance of the large-scale object retrieval system from the coarse-grained level (categories) to the fine-grained level (attributes). Our proposed coarse-to-fine object retrieval (CFOR) system can be robust and resistant to the challenges listed above. To the best of our knowledge, the new main point of our CFOR system is the power of mutual support of object ontology, a local MDNN, and an imbalanced data solver in a unified system. Object ontology supports the exploitation of the inner-group correlations to improve the system performance in category classification, attribute classification, and conducting training flow and retrieval flow to save computational costs in the training stage and retrieval stage on large-scale datasets, respectively. A local MDNN supports linking object ontology to the raw data, and an imbalanced data solver based on Matthews' correlation coefficient (MCC) addresses that the imbalance of data has contributed effectively to increasing the quality of object ontology realization without adjusting network architecture and data augmentation. In order to evaluate the performance of the CFOR system, we experimented on the DeepFashion dataset. This paper has shown that our local MDNN framework based on the pretrained NASNet architecture has achieved better performance (14.2% higher in recall rate) compared to single-task learning (STL) in the attribute learning task; it has also shown that our model with an imbalanced data solver has achieved better performance (5.14% higher in recall rate for fewer data attributes) compared to models that do not take this into account. Moreover, MAP@30 hovers 0.815 in retrieval on an average of 35 imbalanced fashion attributes.

## 1. Introduction

Nowadays, object retrieval is facing some challenges and has some advantages.

Query format plays a very important role in large-scale object retrieval systems. Thus, the query format should be user-friendly and satisfy user requirements in practice.

Two query formats are popular these days: image-based format and text-based format. The text-based query format is being used widely in many searching systems. However, in many cases, it is very difficult to use query text to express the content that human would like to retrieve because words have some limitations in expressing visual information. Instead, a query image is worth more than thousand words; it allows customers to search objects without typing, and the most important thing is that it can retrieve the results based on content. Nevertheless, the limitations of the query image in expressing semantic information could decrease the overall retrieval performance. Thus, the query image and retrieval image with useful related information (regions, categories, fine-grained attributes, etc.) will be the interesting points that we have to focus on to improve the performance of the coarse-to-fine object retrieval system.

Object retrieval systems should meet the requirements of retrieving from large-scale datasets not only at the coarse level but also at the detailed level (or attribute level). For example, in face retrieval systems, facial attribute retrieval is often required. In fashion retrieval systems, fashion attribute retrieval is an indispensable requirement. In person reidentification systems, in the reidentification stage, besides using the global features of the whole human body, attribute vectors of the face and clothes are also being exploited effectively. In crowd attribute recognition systems, the useful attribute set consisted of location, participants, and activities.

Objects often have multiple attributes, and there are methods to retrieve objects at the attribute level from large-scale datasets without manual annotation. In attribute recognition, the traditional methods often waste a lot of time in selecting hand-crafted features for each attribute group during the trial-and-error process but do not always achieve the desired results. In recent years, the deep convolutional neural network (DCNN) has demonstrated high performance in many tasks in computer vision such as detection, classification, recognition, and retrieval. And without exception, the DCNN is also used for attribute learning, with only one network architecture, and the DCNN model can learn to recognize many attributes.

The performance of the DCNN-based attribute learning model will not achieve high rate if the set of attributes plays the same role in the network architecture at the output level and imbalanced data are unresolved. To exploit the inner-group correlations in coarse-grained groups or fine-grained groups, the DCNN often is revised to the deep multitask NN. The performance of classification will be improved if the elements of fine-grained category groups or fine-grained attribute groups could share similar learning features, so the slope of their error surface will become more uniform and the deep multitask learning algorithm can easily reach the global optimum effectively.

Object ontology plays an important role in category classification, attribute classification, and conducting training flow and retrieval flow to save computational costs in the training stage and retrieval stage on large-scale datasets, respectively. Thus, based on our experience in researching objects related to attributes such as face [1], cloth [2], person (reidentification), crowd (monitoring) [3, 4], and fast filters in large-scale object retrieval [5], we would like to introduce an object ontology as a hierarchical semantic tree with three levels: region, category, and attribute levels. The attribute level consisted of visual concepts and specific concepts. Visual concepts support linking common visual attributes to arbitrary objects.

We introduce an object ontology based on popular large-scale standard datasets in science community, so we hope that our ontology can meet the criterion “widely recognized in community.” And for criterion “realization,” we have proposed the local MDNN to support linking object ontology to the raw data. However, if object ontology could not be linked with high quality, it could not function effectively. And we have proposed the imbalanced data solver based on MCC to address data imbalance that has contributed effectively to increasing the quality of linking object ontology to raw data without adjusting network architecture and data augmentation.

We review some typical works based on object ontology, deep multitask neural networks, and imbalanced data solvers to highlight our contributions.

Most of the works only present the set of attributes in the form of item lists or item groups [1, 3, 5–10]. A few works used the terminology “ontology” [11], but to the best of our knowledge, there are not works that present the object ontology in full meaning of regions, categories, and attributes.

In [8], FashionNet handles the challenges as deformation and occlusions by explicitly predicting clothing landmarks and pooling features over the estimated landmarks, resulting in more discriminative cloth representation. The authors do not use the terminology “ontology,” but the DeepFashion dataset is organized based on a hierarchical tree; it is only deployed according to fashion, and it includes a two-level tree: the first level consisted of 50 categories and the second level consisted of 5 attribute groups (texture, fabric, shape, part, and style) (it does not have color attribute). The coarse-grained groups (at the category level) or fine-grained groups (at the attribute level) have the same role in deep neural networks, and the imbalanced data solver has not been considered yet.

In [6], the authors proposed a multitask network to recognize facial attributes, but they did not consider the retrieval problem. They proposed a model to learn multiple attribute labels simultaneously through a single DCNN that supports domain adaption for multitasking. In this case, the task is attribute prediction, and they find a way to simultaneously maximize predictive accuracy of all attributes. However, the authors did not explicitly exploit the inner-group correlations of facial attributes, so the attributes have the same role in the multitask network. The authors did not use ontology to arrange the facial attributes into a hierarchical semantic tree. Imbalanced data are solved based on loss functions associated with each attribute, so their system cannot take advantage of transfer learning.

In [11], the authors presented integration of deep multilevel learning and concept ontology for large-scale visual recognition, but they did not consider large-scale object retrieval. Object ontology consisted of two levels: coarse-grained groups and fine-grained groups. Exploiting the inner-group correlation of attributes and the imbalanced data solver have not been considered yet.

Our idea is to improve the performance of deep neural networks based on object ontology and imbalanced data solvers with inspiration from Gödel's incompleteness theory. This theory shows the limitation of any consistent formal system as well as the limitation of specific methods in solving problems. When the deep network configuration method is not able to create such a large effect as in the early days it took place, it is necessary to integrate object ontology and imbalanced data solvers into deep learning. Based on appropriate interventions in inputs and outputs, we introduce a new method that can help improve the performance of the object retrieval system.

The main contributions of this paper are as follows.

Our proposed unified model consisted of object ontology, a local MDNN, and an imbalanced data solver to improve the performance of the large-scale object retrieval system from the coarse-grained level (categories) to the fine-grained level (attributes).

Our proposed object ontology is a hierarchical semantic tree consisting of three main levels: region, category, and attribute levels. It can support the optimal learning strategy and minimize the effect of semantic gap. It is useful to improve the performance of category classification, attribute classification, and conducting training flow and retrieval flow to save computational costs in the training stage and retrieval stage on large-scale datasets, respectively.

Our proposed local MDNN is inspired by multitask neural networks. It is based on NASNet, ResNet exploiting the local multitask neural network architecture, to improve the performance of category classification and attribute classification and for flexible system updates. The local MDNN supports linking object ontology to raw data and takes advantage of inner-group correlations of categories and attributes. If the inner-group correlations (or intergroup correlations) are exploited, the performance of classification will be improved because the elements of fine-grained categories or the fine-grained attribute group share similar learning features, the slope of their error surface becomes more uniform, and our deep local multitask learning algorithm can easily reach the global optimum effectively.

Data imbalances often occur for large-scale datasets. Data augmentation is almost impossible because each object can have multiple attributes. The solution based on the loss functions, as in [6], may be possible, but it cannot exploit transfer learning. Our proposed imbalanced data solver is inherited from MCC [12] without adjusting network architecture and data augmentation. It is integrated into the local MDNN to improve the performance of category classification and attribute classification, but it can still exploit transfer learning to reduce computational costs in the training stage on large-scale datasets.

Our proposed query format is based on object ontology with semantic information such as regions, categories, and attributes extracted automatically from the query image. Therefore, we can express semantic information from the image to the retrieval process that the traditional methods have not implemented yet.

We experimented on a DeepFashion dataset [8]. The experimental results have shown which architecture is suitable for a specific learning problem from the coarse-grained level to the fine-grained level. They have shown that, with the pretrained NASNet architecture, our local multitask learning (local MTL) framework achieved better performance (14.2% higher in recall rate) compared to single-task learning (STL) in attribute learning. They have also shown that our model considering imbalanced data achieved better performance (5.14% higher in recall rate for fewer data attributes) compared to models that do not take this into account.

The remainder of this paper is organized as follows: Related works are reviewed in Section 2. CFOR is introduced in Section 3. Object ontology is presented in Section 4. Deep local multitask learning framework and imbalanced data solver are presented in Section 5. Retrieving and indexing methods in the CFOR system are presented in Section 6. Experiments and analysis are described in Section 7. We conclude our paper in Section 8.

## 2. Related Works

Our objective is to propose a coarse-to-fine object retrieval system and test its performance on the DeepFashion dataset. Therefore, we briefly review the most recent literature as follows.

### 2.1. Object Retrieval System

Fine-grained object retrieval is supposed to search for similar images that include specific object attributes. It declares a transition model from image retrieval to object attribute retrieval [13, 14]. Specifically, unlike traditional image retrieval systems where queries and results are often coarse (e.g., texts or images), fine-grained image retrieval aims to retrieve semantic information such as categories and attributes. In the fashion field, taking advantages of semantic information, an object retrieval method based on the combination of the global feature with fine-grained attribute information was introduced [8]. Inspired by previous works, we would like to propose a coarse-to-fine object retrieval system which not only takes advantage of the combination of the global feature with fine-grained attribute information but also optimizes the learning strategy based on ontology and resolves the imbalanced data problem by interfering with the output.

In addition to meeting the semantic retrieval results, the object retrieval system must handle large-scale problems to run in real time. In [15], the authors formulate the problem into a mathematical model and derive a closed-form solution with linearithmic time and linear space complexities. In [16], the authors propose fast indexing with a deep convolutional neural network and local geometric constraint model, thanks to the help of locality-sensitive hashing. However, these solutions did not take advantage of the power of GPUs for parallel processing which can significantly reduce feature-matching time and retrieval time. To leverage the support of GPUs, we inherited the search algorithm introduced by Johnson et al. (billion-scale similarity search with GPUs [17]) which is a nonexhaustive similarity search. The search method perfectly suited the proposed CFOR system which further decreased searching time by creating multi-index files based on built-in object ontology.

To clarify the contribution of our CFOR system, we compare it with our main reference DeepFashion [8] in both the offline phase (see Table 1) and online phase (see Table 2).

### 2.2. Data Organization

#### 2.2.1. Object Ontology

At the fine-grained level, the semantic interpretation of a visual scene depends heavily on prior knowledge and experience of the viewer. Vision is an intensive knowledge-based process. Many knowledge-based vision systems have been proposed in the past (VISIONS [18], SIGMA [19], PROGAL [20], MESSIE [21], etc.). The analysis of these knowledge-based vision systems allows us to draw some conclusions: there are three main levels of semantic concepts—the low-level visual concepts, the midlevel semantic concepts, and the high-level semantic concepts [22]. These semantic concepts have been defined and used in many datasets in the form of labels. The most important challenge in image understanding is the semantic gap that has strong effects on system performance. The semantic gap denotes “the inherent difference between the digital representation of an image and the interpretation that the user associates with it” [22]. It is very difficult to teach the computer to directly understand the underlying concepts in an image based on the raw data, but the midlevel semantic concepts could narrow the semantic gap. To narrow the gap between the raw data to high-level concepts in large-scale data, object ontology is proposed by introducing the midlevel semantic concept and its relationships. According to these characteristics of object ontology, it is suitable to apply to fine-grained object retrieval tasks. Maillot et al. [23] demonstrated the advantages of ontology when applied to retrieval tasks.

#### 2.2.2. Attribute Learning

Attribute learning is a backbone of CFOR, and it has strong effects on performance of fine-grained object retrieval. Therefore, attribute learning is considered one of the important parts of the learning strategy.

*(1) Attribute Learning*. This method is used for object recognition systems at the fine-grained level. Unlike learning methods that are used for the high-level concept, attribute learning supports a solution for midlevel semantic concepts or visual concepts which are known to have (more or less) correlations to each other. There are two main different learning methods: single-task learning and multitask learning.

  Single-task attribute learning: in this type, attributes have their own learning model. Therefore, it leads to the number of models equal to the number of attributes. Moreover, each attribute is treated separately, for which the inner-group correlations are not yet exploited.
  Many works are known in the fashion field such as the works [9, 10, 24] using single-task learning for fashion attributes. At that time, there were many challenges in multitask learning. Thanks to this work [25], a shared CNN is defined to pave a way in the final format of the multitask multilabel predictions. Therefore, multitask learning becomes possible.
  Multitask attribute learning: to apply this technique to attributes, samples will be collected by merging given datasets into one with one-hot binary vector demonstration. Like single-task learning, the input will be the image. Despite the output of single-task learning which is a value that describes the existence (or not) of an attribute in an image, the output of multitask learning will be a one-hot binary vector which describes the existence (or not) of a group of attributes.
  Rudd et al. [6] have shown that joint optimization over all attributes outperforms training a single independent network with the same architecture for each attribute, in which the feature space is optimized along with the classifier on a per-attribute basis, both in terms of accuracy and storage, processing efficiency. This result shows that the multitask approach is much more effective in exploiting latent correlations than independent classifiers used to learn them.
  Although multitask learning can yield better performance compared to single-task learning, its critical weakness is that the model cannot be reused when there is any attribute change. A retraining or additional model will be applied when a new attribute is added. Lack of reuse is the reason that multitask learning methods are not flexible for attributes that change frequently. To address these challenges, we propose that local multitask attribute learning be considered a grouping method based on object ontology to improve its reuse.

**Figure 1:**
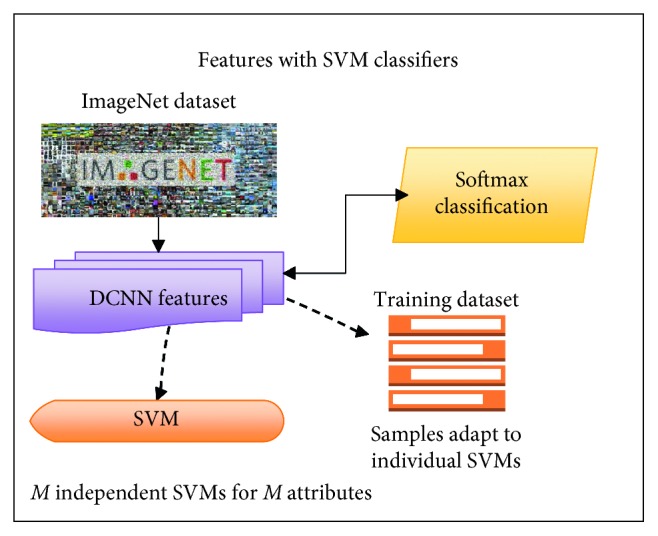
Attribute learning model based on deep features with SVM classifiers.

**Figure 2:**
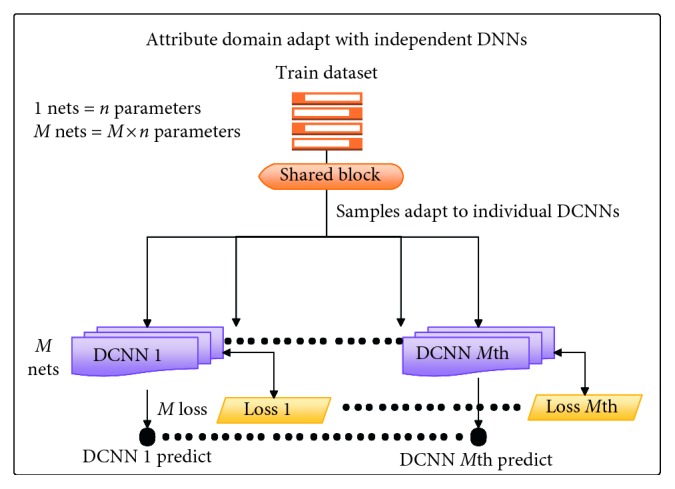
Attribute learning model based on adaptive attribute domain with independent deep convolutional neural networks.

**Figure 3:**
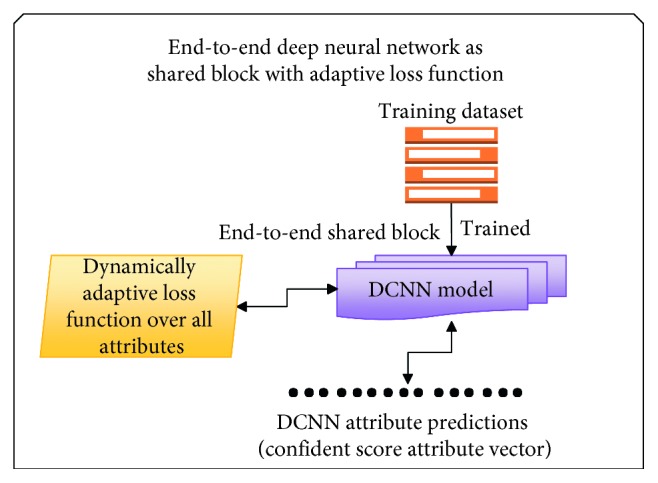
Attribute learning model based on the end-to-end deep neural network as a shared block with adaptive loss function.

*(2) Multitask Learning Methods*. Over the years, there have been many attribute learning methods inspired by multitask learning (see Figures 1–3 for an overview of the method). As far as we know, there are three main attribute learning approaches: features with SVM classifiers, adaptive attribute domain with independent deep neural networks, and the end-to-end deep neural network as a shared block with adaptive loss function.

Besides learning methods, transfer learning is also a significant method that should be focused on for improving performance as well as reducing training time. However, based on the distribution and the size of the dataset, transfer learning can be applied in different tasks and situations.

  Attribute learning model based on deep features with SVM classifiers: these methods inherited the trained features for classification problems and then fed them as inputs into independent SVMs for prediction.
  For example, the initial approach made by Kumar et al. [1] used AdaBoost to select a separate feature space for each attribute and independent SVMs to perform classification. Zhong et al. [26] proposed off-the-shelf CNN feature learning under FaceNet and VGG-16 architecture and then applied an SVM classifier per attribute for classification.
  However, these methods only apply indirectly to multitask learning through global features that are only extracted from a fully connected layer (or other layers which also have high generalization) by a classification model trained on the ImageNet dataset. There is no training on any specific dataset except the ImageNet dataset, so the feedforward network in transfer learning is utilized. Therefore, attribute correlation (including intergroup and inner-group correlations) is not fully exploited yet.
  Attribute learning model based on adaptive attribute domain with independent DCNNs: these methods address the problem with separately trained DCNNs (adaptive attribute domain with independent DCNNs) followed by a group of deep layers (called the shared block). Unlike the previous model, each sample has more than one label, so the output will be an *m*-dimensional attribute vector (*m* is equal to the number of attributes). Each element of the attribute vector represents the existence of the attribute. After passing the shared block to get correlated information, *m* nets with *m* corresponding loss functions are designed to learn *m* attributes. Therefore, each net will predict its corresponding attributes. The backpropagation in each individual net is applied with the same mechanism as a simple classification.
  In these methods, transfer learning can only be applied in each *m* individual net to reduce training time; however, the entire training model is not. Therefore, if the dataset is small and different from the pretrained one, then it may take account of transfer learning without reducing overall performance. In the case of a large and high diversity dataset which needs to fine tune convNet through the entire network, these methods are not a good choice to take advantage of transfer learning.
  In these methods, each model corresponds to an attribute, and inner-group correlation is not given an advantage. However, as in [2], these methods can make a good extraction for intergroup correlations between attributes.
  This work [7] shows that joint multitask attribute learning can achieve better performance compared to deep feature-based attribute learning. Although it improves the state-of-the-art attribute recognition accuracy, it consumes a lot of computer resources and training time depends on the number of attributes. This work succeeded in face attribute recognition; it is one of the initial methods applied in this multitask learning method. They used the AlexNet network modified as a shared block and VGG-16 for each individual attribute.
  In the fashion field, in [2], attributes were divided into smaller groups, and a pretrained CNN model (based on ImageNet) for each group and a shared latent matrix between all CNN models are used. For face attributes, the study [7] used shared feature learning at an early stage for all the attributes followed by category-specific feature learning for heterogeneous attribute categories. Although these methods are on different fields, they have the same main idea—attributes are divided into smaller groups or smaller categories which can exploit intergroup correlations and inner-group correlations between attributes.
  Although these methods outperform the attribute learning method based on deep features, they consume computer resources because of the expansion of the number of parameters according to the expansion of the number of attributes.
  Attribute learning model based on the end-to-end deep neural network as a shared block with adaptive loss function: this approach uses an end-to-end architecture as a shared block between attributes. To adapt, the objective function reweighs each part of the loss associated with each attribute. This approach can extract inner-group correlations between attributes and can easily configure the architecture or input data to learn intergroup correlations.
  This work, the mixed objective optimization network (MOON) architecture with the loss of domain adaptive multitask DCNN proposed by Rudd et al. [6], is an example of this group of method. The MOON learns to balance its multitask output predictions with reduced training and storage costs while still producing better accuracy compared to independently trained DCNNs. Mixed objective dynamic adaptive loss function plays an important role in solving imbalanced data problems. As in [6], a joint optimization with respect to all attributes achieves the performance superior to the first approach (features with SVM classifiers).
  Although these approaches provide a better solution for training resources as well as imbalanced data problems, transfer learning is difficult to adapt because the architecture and loss function have been modified to support multitask, multioutput, and imbalanced data problems.  Table 3 shows the main differences in the contribution of the three introduced multitask learning methods as well as our proposed deep local multitask learning, which will be mentioned in following sections, in different criteria.

*(3) Imbalanced Data Problem*. Imbalanced data are the problem in machine learning in which the class distribution is not uniform between the classes. Usually, they are composed of two types of classes: the majority classes (positive) and the minority classes (negative). Recent research in machine learning shows that using an uneven distribution of class examples during learning can cause learning algorithms with misleading performance (bias). It means a classifier with high accuracy in the majority, but it gives poor accuracy in the minority class. In the case of attribute learning, an imbalance occurs if the number of instances in some attributes varies significantly in quantity compared to other attributes. To deal with this situation, in general, adjusting the distribution of classes is an essence of many popular methods to handle imbalanced data problems.

  Data sampling: sampling-based methods such as upsampling, downsampling, or data augmentation are considered to be a solution for imbalanced data problems. In addition to making data more balanced, they can help reduce training time (downsampling) or make the learning process more efficient (upsampling). The best approach we know is SMOTE [27] which can solve the situation by automatically generating additional data (upsampling) based on the original dataset. However, these methods increase overfitting when training (upsampling) or losing (downsampling) data. Data augmentation is proved to be robust in dealing with imbalanced training data [28]. However, this method takes up a lot of training resources, and it is difficult to find a proper augmented dataset which is large enough to train. And it is very difficult (or impossible) to augment data to balance the attributes in datasets because each object usually has many attributes.
  Architecture, loss function, and metric configuration: other methods exploit network architectures, loss functions, or metrics to address the imbalanced data problem when training. The methods (at the algorithm level) enhance the existing classifier by adjusting algorithms to recognize the smaller classes. Internal techniques provide general solutions for the imbalanced data problem because these are not specific to particular problems. This work [6] is an example for dealing with the imbalanced data problem in attribute learning by creating a mixed objective dynamic adaptive loss function and solving the problem internally. These approaches show better performance compared to data sampling; however, they are often difficult to implement as well as configure in the future. Therefore, they are not always the best choice in dynamic retrieval systems in which the attributes have a large variety.
  Threshold and output-based configuration: instead of generating more data or making changes in the model, these methods find the best thresholds based on output. The essence of these methods is to use scores that show the probability to indicate which test sample is a member of a class in producing several learners by changing the threshold for class members. These methods are particularly effective in resolving imbalanced data problems without changing the configuration in the model. Moreover, they also do not reduce data or increase overfitting. SVM is proposed to find these thresholds [12]. However, Boughorbel et al. [12] proposed Matthews' correlation coefficient (MCC) [29] to deal with imbalanced data in classification. Although SVM shows better performance, MCC consumes less resources and processing time compared to it [12].
  Inspired by studies [6, 29] and based on the methods of many other researchers, we found a solution for multitask learning that is suitable to retrieval systems using the end-to-end DCNN for training and MCC for estimating thresholds to get final outputs.

*(4) Deep CNN Architectures*. They show their performance for hand-crafted features (SIFT [30], HOG [31] or color histogram, LBP [32], etc.) on large-scale datasets. Hence, the popular deployment in [33, 34] along with the usage of pretrained CNN models on the ImageNet dataset [33] makes it easier to fine tune various DCNN architectures [35, 36] for multiple visual datasets. Fine-grained object recognition systems have to deal with a large number of images on large-scale datasets. Thanks to transfer learning, we can reduce training time. However, transfer learning which is applied in some available architectures is not designed to solve imbalanced data problems. Thus, the overall performance will decrease when encountering this problem.

Pretrained VGG and AlexNet are used in multiple attribute learning systems which can be found in FaceNet [26] and Han et al.'s study [7] for facial attribute learning, respectively. In the fashion field, Abdulnabi et al. [2] use the ImageNet pretrained CNN model for solving multitask attribute learning. However, there are many high-performance architectures (like ResNet [35] which can handle well with bias, gradient vanishing or NASNet [36] which can automatically build a model based on data) which passed beyond human ability in ImageNet classification but have not been applied yet. In our proposed method, these architectures will be put into use.

## 3. Materials and Methods: CFOR System

The CFOR system is very complicated but easy to understand. We focus on the main points of the CFOR system.

**Figure 4:**
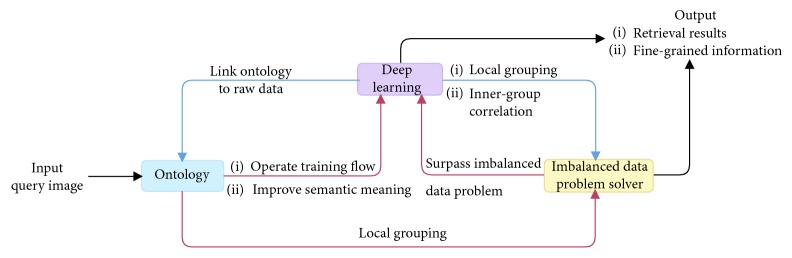
Synthesis of object ontology, deep learning, and imbalanced data problem solver in the CFOR system.

CFOR is an object retrieval system integrated by object ontology, a local MDNN (NASNet and ResNet), and an imbalanced data solver (MCC) to improve the performance of the large-scale object retrieval system from the coarse-grained level (categories) to the fine-grained level (attributes) (see Figure 4).

**Figure 5:**
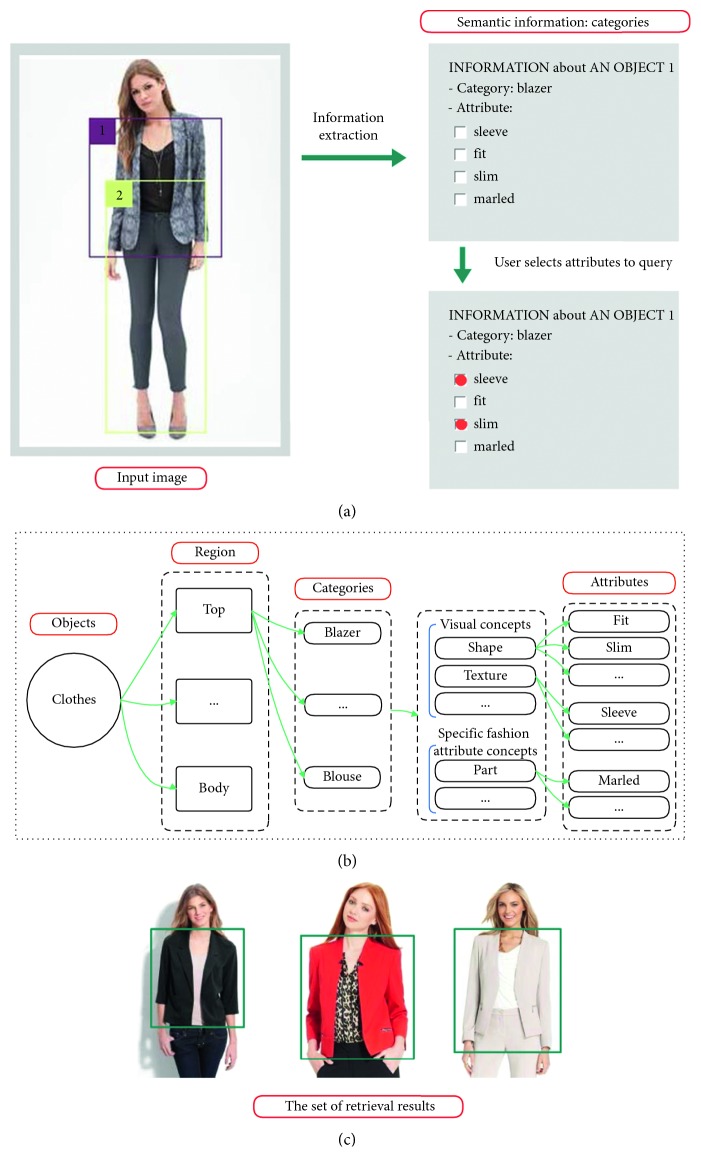
(a) Extracting regions, categories, and attributes from a query image with trained models of the CFOR system. After that, users can use this semantic information to reduce the searching space. (b) Fashion ontology used to retrieve. (c) Retrieval results.

1. *Query Image*. For traditional content-based image retrieval systems, with query images, one is just able to retrieve the images ranked on visual similarity to query image. It is very difficult (or impossible) for users to provide semantic information to the system based on query images. But the interesting thing is that, in our CFOR system, this challenge has been solved. The semantic information of the query image is extracted automatically by the category and attribute classification system, and users can use the extracted semantic information during the retrieval process.
2. An example is how users can query “Asian face” with only a query image; here, “Asian race” is semantic information. The traditional retrieval methods cannot meet this requirement because of the curse of semantic gap. And the CFOR system can recognize “Asian race” and use it to retrieve. Another example for “Fashion” object based on our CFOR system is described in Figure 5. From the query image, based on fashion ontology, the detector quickly identifies the region (Top and Bottom; see Figure 5). After that, the user selects the region (Top; see Figure 5); the CFOR system quickly identifies the category related to the Top region (category: Blazer). Later, specific concepts and visual concepts are extracted according to Blazer, and users can select some of them (or all of them) to retrieve. For user-friendly interaction, only extracted regions, categories, and attributes are shown. Other information such as global deep features, attribute vector, ontology, or group of attributes which are used as searching input of the system will not be displayed. In such a way, users can order the CFOR system at the semantic level, and they can achieve the results that match both the content and semantics of the query image.

The CFOR system is organized into two main phases: offline phase and online phase.

**Figure 6:**
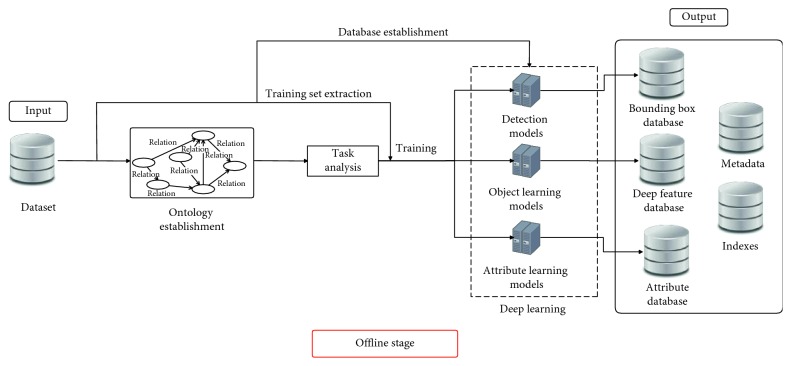
Offline stage of the CFOR system.

**Figure 7:**
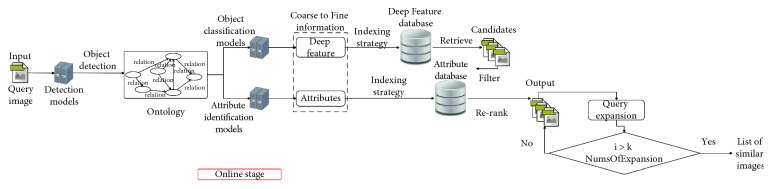
Online stage of the CFOR system.

1. *Offline Phase*. This phase is designed to generate object ontology, database, indexing file and region detection model, category classification model, and attribute classification model.
2. Object ontology is designed manually based on professional experience and public dataset for the community. It is organized into a hierarchical semantic tree with three main levels: region level, category level, and attribute level.
3. The database is generated to store the preextracted features, regions, categories, and attributes of all images in the dataset. It supports to reduce the online retrieval time and provides the necessary semantic information for each retrieved image.
4. The indexing file which is created to support fast mapping in the online phase of the CFOR retrieval system is the key to perform the retrieval task at runtime.
5. Regions, categories, and attributes are learned automatically based on the local MDNN. Detection models and classification models are created to extract or predict semantic information of the query image and dataset such as regions, categories, and attributes.
6. *Online Phase*. This phase of the CFOR system is designed to run the retrieval process including object detection, semantic information extraction, and query expansion and retrieval.
7. In the object detection stage, we use the trained object detector to detect objects in the query image. In the semantic information extraction stage, the built-in object ontology and classification models are used for extracting the necessary semantic information of each identified object. The extracted semantic information and deep global features of each detected object passed through the searching system along with the indexing file to quickly compute the score between the query object and the sample in the database. Retrieval is applied to rank and export the most similar images to the query object and their relevant information. Query expansion is optional and used to increase the retrieval performance with a trade-off for retrieval time.
8. The power of mutually supporting object ontology, local MDNN, and imbalanced data solver in the CFOR system: Figure 4 shows the operation of the CFOR system with the interaction of the three main modules object ontology, a local MDNN, and an imbalanced data solver to optimize the learning strategy and improve the overall retrieval performance on large-scale datasets.
9. Object ontology supports conducting the training flow (with a local MDNN) and retrieval flow (from the coarse-grained level to the fine-grained level) to save computational costs in the training stage and retrieval stage on large-scale datasets. Training flow also paves a way for applying transfer learning which may improve the convergence rate of deep networks. Object ontology which could transform the global imbalance of data into local imbalance of data based on fine-grained groups makes the imbalanced data problem easier to deal with.
10. Deep multitask NN supports to link the object ontology to the raw data effectively at the category level and attribute level by exploiting inner-group correlations and intergroup correlations. The object ontology supports to update the system at the local level with parallel processing based on the local MDNN. Therefore, CFOR is updated in a flexible manner on large-scale datasets with many variations.
11. And the proposed imbalanced data solver based on MCC which addresses data imbalance has contributed effectively to increasing the quality of object ontology implementation without adjusting network architecture and data augmentation.
12. Algorithm and demonstration of the CFOR system: an online phase and offline phase (Figures 6 and 7) are used to analyze tasks in the CFOR system. These phases will be demonstrated in detail in this section. The retrieval algorithm in the CFOR system is described at the offline phase (see Algorithm 1 and Section 3.1) and online phase (see Algorithm 2 and Section 3.2). Besides, the CFOR system can be put into use as a general solution for retrieval. To evaluate the performance of the proposed system, fashion objects with attributes are selected in experiments.

### 3.1. Offline Phase

This phase consisted of three substages:

 
*Object Ontology Establishment Stage*. This stage defines fashion ontology to control the training flow as well as the online retrieval flow which serves as a bridge between high-level concepts (objects and categories), midlevel concepts (attributes), and raw data.
 
*Learning Stage*. This stage exploits deep networks with transfer learning in dealing with the specific tasks including object part learning, category learning, and attribute learning.
 
*Storing and Indexing Stage*. This stage defines a way of storing data as well as making the index list to reduce retrieval or searching time.

From the offline phase, in this section, inherited from previous state-of-the-art methods, we will mention about object part extraction, transfer learning, and its role in the retrieval system as well as data storing. These modules are highly generalized to any object. Other issues including ontology, attribute learning, network architecture, and indexing strategy will be detailed in the following sections. In addition, the offline phase of the CFOR system is also introduced technically in Algorithm 1 according to Figure 6.

#### 3.1.1. Loss Function

This function inherited the current state-of-the-art ResNet for classification, and cross entropy loss function is applied for multiclass classification in the category classification model and attribute classification model.

For attribute multitask classification models, the loss function is described as follows:

(1) $$\begin{array}{c} ℒ\left(\theta \right)=-\frac{1}{n}\overset{N}{\underset{i=1}{\sum }}\left[y_{i}\;\log\left(\hat{y_{i}}\right)+\left(1-y_{i}\right)\;\log\left(1-\hat{y_{i}}\right)\right]\\ =\;-\frac{1}{n}\overset{N}{\underset{i=1}{\sum }}\overset{M}{\underset{j=1}{\sum }}y_{i}^{j}\;\log\left({\hat{y}}_{i}^{j}\right)+\left(1-y_{i}^{j}\right)\;\log\left(1-{\hat{y}}_{i}^{j}\right),\; \end{array}$$

where ${\hat{y}}_{i}^{j}\;$ is the prediction for a sample, *y*_*i*_^*j*^ is the corresponding ground truth, *N* stands for the number of samples, and *M* stands for the number of attributes.

#### 3.1.2. Technical Details

In Algorithm 1, object ontology which is described in detail in Section 4, is designed manually based on professional experience and public dataset for the community. It is organized into the hierarchical semantic tree with three main levels: region level, category level, and attribute level. Regions, categories, and attributes are learned automatically based on the local MDNN. The DeepFashion dataset [37] has been manually annotated, and our contribution follows fashion ontology. Besides, to clarify Algorithm 1, the used functions will be described as follows:

- extract_predicates(dta): in a rich-annotated dataset, e.g., DeepFashion [8], a sample image can be annotated by many labels in different fine-grained levels. For each fine-grained level, the function is used to extract the unique possible labels of samples and then store these labels into a corresponding array. For example, in the DeepFashion dataset [8], Top, Bottom, and Body are unique labels belonging to one fine-grained level, and thus, they are stored into one array. Similarly, fabric, shape, part, style, and texture labels belong to one fine-grained level and are stored into one array.
- build_ontology(predicates, prior): this matches the extracted level and its labels from each predicate array into the corresponding stage of the general ontology, i.e., prior. For example, Top, Bottom, and Body belong to one level which is matched with the region stage of the ontology. After the matching is finished, all other unused stages are eliminated from the general ontology to generate the adapted ontology, e.g., fashion ontology.
- extract_state(onto): from the built ontology, all stages and their labels are searched and stored into arrays which will be used to reconstruct the data. For example, the region stage array contains three classes, and the category stage array contains 50 classes.
- extract_nes_dta(dta, state, onto): based on the stage and the classes extracted from the “extract_state” function, the whole DeepFashion dataset will be split. Only samples having the labels belonging to the stage are stored as the training set of that stage in the ontology. For example, with the region classification model, only samples labelled Top, Body, or Bottom are used for training.
- classifyModel(architecture, state_dta): in the DeepFashion dataset [8], based on ontology, there are four classification models: region classification model and category classification model for the Top region, Body region, and Bottom region. These models are retrained from the ImageNet dataset [33] using ResNet-101 [35].
- multitaskModel(group_state_dta, architecture, Matthrew_coef = True): for each group state in terms of the fine-grained attribute level, a multitask classification model is built, e.g., fabric attribute group classification model and style attribute group classification model. These models are retrained from the ImageNet dataset [33] using NASNet v3 [35]. Besides, the attribute learning and the usage of MCC are mentioned for an imbalanced data solver and described clearly in Section 5.
- indexing(state_sta): indexing files are created that will be used for run-time retrieval. The method is based on the nonexhaustive compressed-domain search with GPU, which is described clearly in Section 6.
- build_storage(onto, states): storage structures are automatically created based on built-in object ontology and extracted states. The storage structures are described clearly in Section 3.1.5.
- infor_extract(states, dta, onto, classifyModels, multitaskModel): for each sample in the database, all attribute learning models trained in “multitaskModel” function are run and then all possible attributes which are higher than thresholds are extracted. For more details, see Section 5, Algorithm 4, and Algorithm 5 for how thresholds are identified.
- feat_extract(dta, onto, classificationModels, multitaskModel): for each sample in the database, the features of the pre-softmax layer in four models trained in “classifyModel” function are obtained.
- structure(storage, feat_dta, info_dta, indexFiles): the database is automatically built based on extracted features, extracted information, index, and storage structure. The storage structure is described clearly in Section 3.1.5.

#### 3.1.3. Object Part Extraction

For the aforementioned reasons, foreground objects should be extracted from background regions efficiently and accurately before entering the retrieval step. The target of object extraction is to filter the necessary specific subjects. This also improves the efficiency of the following modules as well as increases the overall system performance. There are many successful object detection methods [31, 38, 39]. Among them, YOLO [39] shows the state-of-the-art results. In our system, we inherited the successful software YOLO (version 3.0) to identify fashion items.

#### 3.1.4. Transfer Learning

Transfer learning is one of the best methods to reduce training time, especially with complicated architectures such as ResNet or NASNet. The key issue is the initial parameters. In the first step of the training process, we have to generate these parameters with some unsupervised learning methods. However, the initial one will be far from the optimal one. In transfer learning, we will reuse the trained parameters on a large and diverse dataset (such as ImageNet dataset [8]). By this way, our training process will be easier to meet convergence. Thus, it reduces the training time.

Transfer learning can be applied in different ways based on the size of the dataset and data similarity. There are four scenarios in total. First, if the data size is small while data similarity is high, we use the pretrained model as a feature extractor. Second, if the data size is small and data similarity is low, we freeze the top layers and train the remaining layers of the pretrained model. Third (ideal situation), if the data size is large and data similarity is high, we can retrain the model by using the weights initialized in the pretrained one. Fourth (worst situation), if the data size is large and data similarity is low, transfer learning cannot be applied, and we have to train our model from scratch. In our fashion example experiments, while DeepFashion [8] is a large dataset and ImageNet (dataset used for transfer learning) is a high diversity one, we can use all of the initialized weights from the pretrained model.

According to our approach, transfer learning will be applied in region, category classification as well as attribute learning along with ResNet and NASNet architectures, respectively. It can also be used in global deep feature extraction to improve the overall retrieval performance.

#### 3.1.5. Data Storing

**Figure 8:**
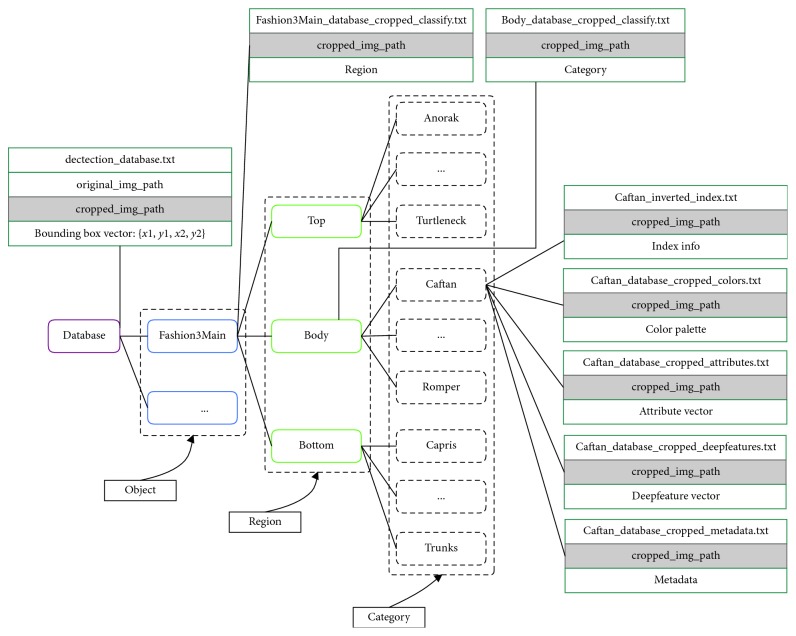
An example of the storing structure.

Features extracted from the category classification task and attribute learning will be stored in a hierarchical semantic tree based on object ontology. All features belong to a leaf of object ontology and will be stored in one file. In case of the expansion of large-scale data, the mentioned files can be indexed and split with a corresponding mapping key for each image. The folders will be organized based on object ontology in which each name corresponds to each concept. To clarify, data storing for the proposed ontology is defined as follows (see Figure 8 for an example of data storing):

- All files are stored in a folder named “database,” which is denoted as the “Object” node.
- Based on ontology, “Object” node contains 3 nodes at the “Region” semantic level. Thus, we have 3 smaller folders: “Top,” “Body,” and “Bottom.”
- At the next stage of ontology, we have the “Category” semantic level. Thus, we have 50 folders representing all nodes of “Category.”
- Finally, we have the “Attribute” semantic level standing for the leaf node state in ontology. At this state, all features belong to the same “Region” and “Category” and are stored in one file.

### 3.2. Online Phase

Algorithm 2 shows the online phase of the CFOR system corresponding to the demonstration in Figure 7.

#### 3.2.1. Technical Details

To clarify Algorithm 2, the used functions will be described as follows:

- detector(imgQuery): an object in an image is automatically detected by using a trained detector. In this function, we inherit the successful software YOLO (version 3.0) to identify fashion items. Besides, the items identified are also refined by the region identification model, which is trained by “classifyModel” function in Algorithm 1.
- infor_extract(states, obj, onto, classifyModels, multitaskModel): for each query object, all attribute learning models trained in function “multitaskModel” and coarse classification models in function “classifyModel” in Algorithm 1 are run. We extract the region ⟶ category ⟶ attributes and necessary features for each stage of the ontology.
- query_expansion(infor, feat): query expansion based on the mean vector is used for reranking retrieval results. See Algorithm 3 for the details of query expansion.
- compute_sim_score(database, infor, feat): for each pair of features, asymmetric distance is used to measure the dissimilarity distance between the query and the sample in the database (see Section 6 for more details). The computation is made parallel by using indexing files obtained from Algorithm 1 for all samples in the database.
- ranking(score_list, database, top_*k*, GPU_search = True): based on the score between the query and all samples in the database obtained from function “compute_sim_score,” ranking is applied; smaller is better.
- retrieval(indexes, score_list, database, GPU_search = True): the retrieval process contains 3 steps including feature retrieval, fine-grained retrieval, and query expansion. For global retrieval, global features of the query object obtained from function “infor_extract” and the features of samples in the database are passed to function “ranking” to get 1^st^ top-*m* retrieval results. For fine-grained retrieval, attribute features (see Section 5 for more details) of the query object obtained from function “infor_extract” and the features of samples in 1^st^ top-*m* retrieval results are passed to function “ranking” to get 2^nd^ top-*k* retrieval results. For query expansion, the mean vector is computed from 2^nd^ top-*k* retrieval results, and each feature of 2^nd^ top-*k* retrieval results is passed to function “ranking” to get final top-*k* retrieval results, i.e., query expansion-based reranking.

As described in Figure 7, the online phase of the CFOR system contains three stages which will be put into use in real time. They are given as follows.

#### 3.2.2. Prediction Stage

This stage will take advantage of object ontology and classification models obtained from the offline phase and then makes predictions from coarse to fine for each query image:

(2) $$\begin{array}{c} I_{q}\left(x,y\right)\;\;⟶O_{I_{q}}\left(M:\left[0,\;\;255\right]\;\times \;\left[0,\;\;255\right]\;\times \;\left[0,\;\;255\right]⟶\left[0,\;\;255\right]\;\times \;\left[0,\;\;255\right]\;\times \;\left[0,\;\;255\right]\right),\\ O_{I_{q}}\left(x,y\right)\;⟶\;R_{I_{q}}\left(M_{P}:\left[0,\;\;255\right]\;\times \;\left[0,\;\;255\right]\;\times \;\left[0,\;\;255\right]\;⟶\;R^{p}\right),\\ R_{I_{q}}=\left(\mathrm{regio}n^{1},\mathrm{regio}n^{2},\mathrm{regio}n^{3},\ldots ,\mathrm{regio}n^{p}\right),\\ R_{I_{q}}^{t}\left(x,y\right)\;⟶\;C_{I_{q}}^{t}\left(M_{C}:\left[0,\;\;255\right]\;\times \;\left[0,\;\;255\right]\;\times \;\left[0,\;\;255\right]⟶R^{c}\right),\\ C_{I_{q}}^{t}=\left(\mathrm{cat}e^{1},\mathrm{cat}e^{2},\mathrm{cat}e^{3},\ldots ,\mathrm{cat}e^{c}\right),\\ C_{I_{q}}^{t}\left(x,y\right)\;\;⟶\;A_{I_{q}}^{t}\left(M_{A}:\left[0,\;\;255\right]\;\times \;\left[0,\;\;255\right]\;\times \;\left[0,\;\;255\right]\;⟶\;R^{a}\right),\\ A_{I_{q}}^{t}=\left(\mathrm{att}r^{1},\mathrm{att}r^{2},\mathrm{att}r^{3},\ldots ,\mathrm{att}r^{a}\right), \end{array}$$

where **I**_**q**_(**x,y**) is the query image, **O**_**I**_**q**__ is the object identified (demonstrated as an object bounding box), **R**_**I**_**q**__ is the object region, **R**_**I**_**q**__^**t**^(**x,y**) is the object with region information, and **C**_**I**_**q**__^**t**^ and **A**_**I**_**q**__^**t**^ are the object category and object attribute, respectively.

Fine-grained information in terms of regions, categories, and attributes provides more options for a customer to give a full semantic query. The object will be predicted from coarse to fine. In turn, the region, category, and attribute will be predicted based on object ontology and a local MDNN. The object retrieval system uses extracted semantic information as the category and attribute to search in detail.

#### 3.2.3. Dissimilarity Measuring Stage

This stage will take advantage of the database as well as the indexing file from the offline phase and a dissimilarity measure to get scores and then rank, rerank, and release retrieval results for each query image. This stage is based on the dissimilarity measure between attribute vectors of query images and database images:

(3) $$\begin{array}{c} \mathrm{RI}\left(I_{q}\right)=\left\{I_{1}^{q},I_{2}^{q},I_{3}^{q}\;\mathrm{,\ldots ,}I_{k}^{q}\;\;|\;d\left(A_{I_{q}},A_{I_{i}^{q}}\right)\mathrm{<d}\left(A_{I_{q}},A_{I_{\mathrm{i+1}}^{q}}\right)\right\},\\ I_{i}^{q}\in D;0\;\leq i<k-1. \end{array}$$

Based on combination of *K*-nearest neighbour search in terms of L2 distance and asymmetric distance computation (ADC will be mentioned in Section 6), we take advantage of parallel processing by GPU through the Faiss method [17] to compute the distance from the query image to the necessary one in the database. The distance which is also called the score of each image in the database is then sorted to rank the dissimilarity. The smaller the score of the image, the more similar the query. Based on the number of retrieval images required or thresholds, we will have an appropriate cutoff in the score as well as the number of retrieval images. This kind of measurement is used to compute distance for both deep features vectors and attribute vectors.

#### 3.2.4. Query Expansion Stage

Query expansion is a technique that can help gather additional relevant information from the input to increase retrieval performance. The information can be relevant images, additional features, description, etc. based on the query expansion algorithms and data. In this stage, we would like to take advantage of the previous retrieval results and then expand the query by using the mean vector to rerank and get reranked retrieval results to improve retrieval performance.

Query expansion based on the mean vector is chosen among many methods mentioned in [40–42] because of its trade-off in speed and performance and also suitable for large-scale datasets. When extracted features represent a query image passing through the CFOR system, retrieval results can contain outliers due to the limitation of similarity mapping between input features and samples in the database. By applying Algorithm 3, the mean vector computed from features of retrieval results and the features of input help reduce the bias between different considered features. Thus, the CFOR system can eliminate unrelated features; that is, retrieval features have high gap from the mean vector features, which helps reduce outliers and rise the precision score.

Query expansion based on computing mean vector is performed very fast, and it can take advantage of the Faiss similarity searching method [17] as well. Query expansion can remove outliers, thanks to the statistic essence of the mean vector.

## 4. Fashion Ontology: CFOR System Testing in Fashion

In this section, we will mention about ontology, fashion ontology, and its related information and present the contributions of object ontology to the CFOR system.

### 4.1. Ontology Definition for CFOR System

As mentioned by Guarino in [43], ontology is defined as a “formal, explicit specification of a shared conceptualization.” Most ontologies are described as a group of concepts followed by their relative structure, which can help describe and support information for a domain. A completed ontology is supposed to have a group of concepts (C), a corresponding set of relations (R), and finally axioms. Also, as in [22], ontologies provide some main advantages:

- Describe the domain knowledge in the form of the semantic hierarchical tree including the nodes that are concepts that can be called by words or phrases
- Support narrowing the semantic gap in many tasks in computer vision and other disciplines
- Achieve important improvements in software engineering: flexibility, reliability, specification, and reusability
- Have potential to support solving multitask problems

The proposed ontology should meet the following two basic requirements:

- Widely recognized by the community
- Ability to be formalized by mathematical expressions (ability to be digitized)

In our approach, we use ontological engineering for communication and information sharing between different data abstraction levels involved in image fashion retrieval, detection, and information tagging.

Object ontology consisted of two main levels: coarse-grained level and fine-grained level.

- Object ontology at the coarse level consisted of regions, categories, or any kinds of high-level concept ones which can use global features extracted by the deep network. These global features can be used for similarity retrieval. However, deep features are treated as black boxes, so no semantic information can be shown out for supporting customers in their searching process.
- Object ontology at the fine-grained level consisted of the object's attributes that can be used to describe the object in detail.

**Figure 9:**
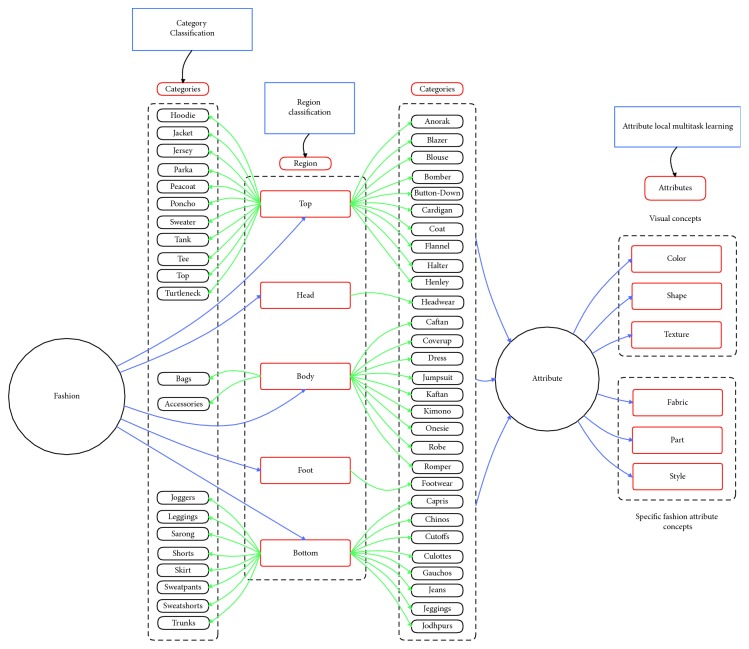
Fashion ontology in general and a version of ontology for clothes.

Object “Fashion” is described in our experiment. Fashion ontology is created by prior knowledge and information on the DeepFashion dataset [8] and ontology definition introduced by Guarino [43] (see Figure 9 for fashion ontology).

The three most important semantic levels of the developed fashion ontology are as follows:

- Regions (a region, e.g., for clothes: Top, Bottom, and Body)
- Categories (consisted of specific objects linked to the region, e.g., for Body: dress, robe, etc.)
- Attributes (describing visual fine-grained concepts, e.g., fabric: denim, fur, etc.)

To focus on the necessary main points, we only investigated the object fashion at three regions (Top, Body, and Bottom), some main categories related to three regions, and their attributes.

**Figure 10:**
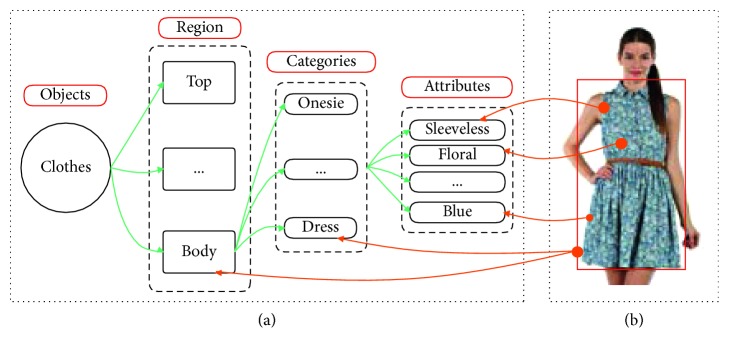
An example of a relationship between the query image and semantic information from the coarse-grained level to the fine-grained level of the fashion ontology.

In the CFOR system, a query image will be fed into the system from the coarse level based on object ontology to determine the region and category of the corresponding object. Then, each object with the coarse information will go through fine-grained concept ontology to identify attributes. After the corresponding object gets all of the needed information, it will get through the indexing step and compute similarity distance step to help find out a similar image in the database with a ranked score. Ranked score is the sum of the similarity score of global features extracted from the category classification task and the similarity score in attribute learning between the query image and the target database image (see Figure 10 for more details).

### 4.2. Fashion Object Ontology

In this section, we propose the fashion object ontology. In the fashion field, we divide semantic fashion concepts based on the region (Region). For each region, we will have a more detailed ontology based on categories and attributes. For supporting experiments in the DeepFashion dataset [8], we expand the fashion ontology in the “Clothes” branch (see Figure 9). It is important to note that the proposed ontology is not application dependent and should be considered as an extensible basis.

Fashion object ontology includes multiple levels of concepts. Between each level is a set of relations to describe their relationship. There are two main relations:

- “part of”: the relation is used to specify the concepts are parts of the main concept
- “has a”: the relation is used to describe the main concept in detail

**Figure 11:**
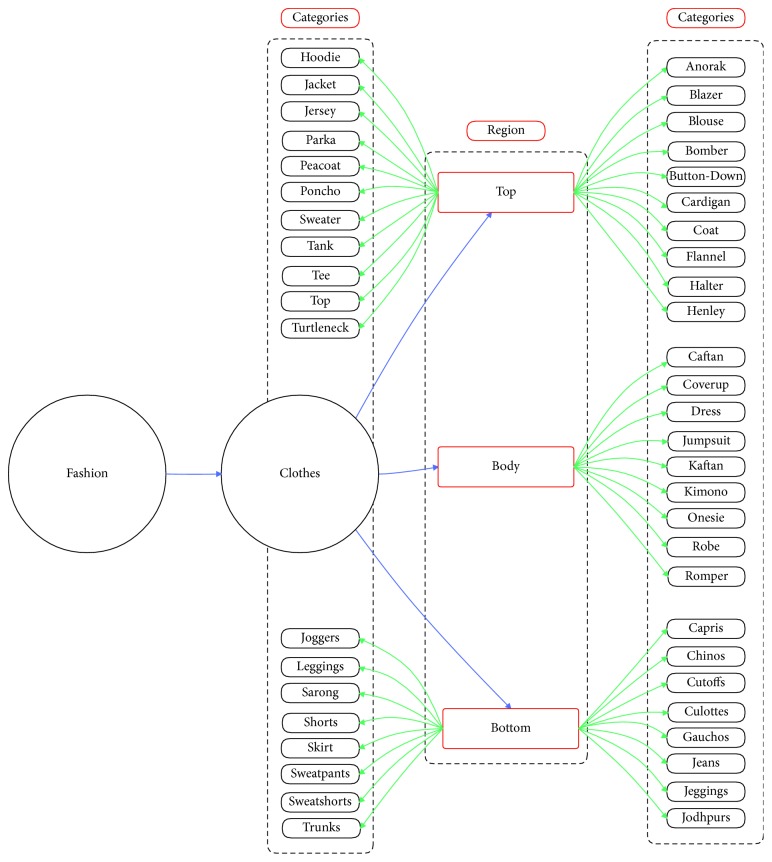
Excerpt from the “Clothes” taxonomy defined in the fashion ontology.

In this research, we focus only on Clothes branch to make fair comparisons with other methods. Clothes taxonomy has 50 different categories. A cloth region taxonomy has been defined (see Figure 11), arranging all cloth categories into a hierarchy, the first level of which corresponds to the most general region of clothing. 3 main regions were defined:

- Top (e.g., tee and tank)
- Bottom (e.g., skirt and jeans)
- Body (e.g., dress and robe)

### 4.3. Fine-Grained Object Ontology

**Figure 12:**
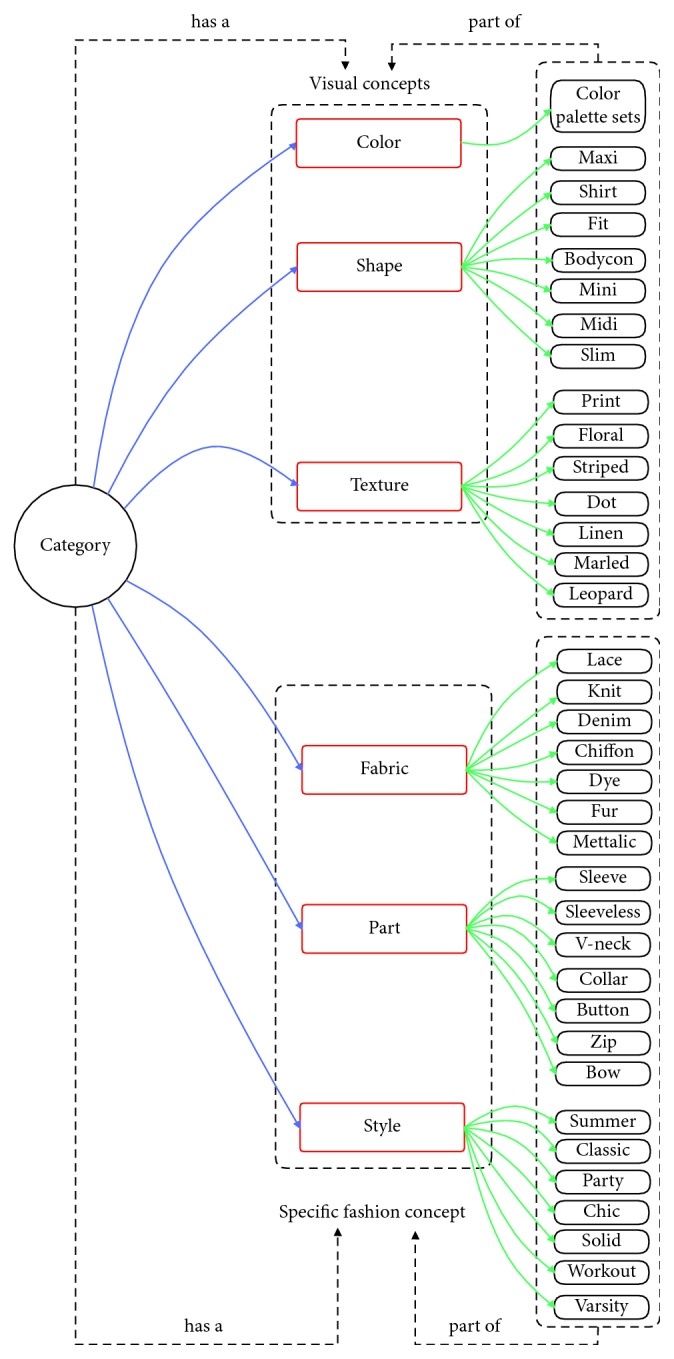
Fine-grained group at the attribute level.

Fine-grained object ontology is used to describe objects at the attribute level. Semantic information such as attributes can be useful for a customer to retrieve (see Figure 12). It is important to note that the proposed ontology is not application dependent and should be considered as an extensible basis.

Cloth attributes are defined on different levels—some attributes are popular in all cloth regions (e.g., color) and some attributes are reserved to only certain regions or categories. We have structured ontology in two main parts; each part of this ontology is detailed in the next sections:

- Specific fashion concepts—related to particular characteristics of clothes (fabric, part, and style).
- Visual concepts—related to the popular visual characteristics (color, shape, and texture); they are not reserved only for fashion.

In [6], Rudd et al. have proved that a multitask learning-based model shows better performance in accuracy compared to a combination of single-task learning-based models in face attribute prediction. This method can be applied to fashion attributes and also shows good results. However, unlike face attributes which have a limitation in quantity, there are a large variety of fashion attributes. This method can lead to difficulties in expanding system (e.g., training and storing). Based on the levels of fashion ontology, we can apply local multitask learning to attribute learning more flexibly. The explanation is also given in the next sections.

#### 4.3.1. Visual Concepts

**Figure 13:**
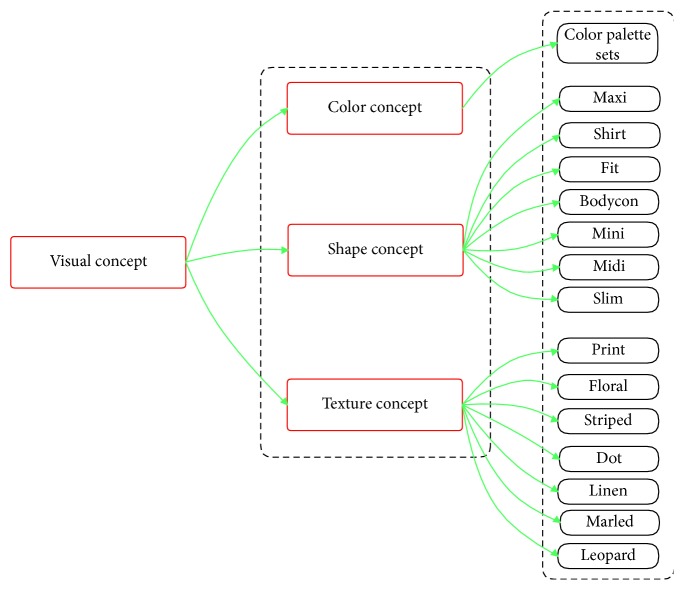
Visual concept ontology.

Visual concepts consisted of shape concepts, texture concepts, and color concepts. These visual concepts are usually stable and have a limitation in quantity. Thus, it leads to the fact that we can use local multitask learning to solve the attribute prediction problem. Moreover, models trained in this way can take advantage of inner-group correlations to improve performance (see Figure 13 for visual concepts).

*(1) Shape Concepts*. This part of ontology has been inspired by results from the DeepFashion dataset (Liu et al. [8]). In category and attribute prediction benchmark, there are a total of 180 shape attributes, and we use all of them for shape concepts (see Appendix for more details). However, we experiment in smaller version for shape concepts such as maxi, shirt, fit, bodycon, mini, midi, and slim.

*(2) Texture Concepts*. This part of ontology has been inspired by results from the DeepFashion dataset (Liu et al. [8]). In category and attribute prediction benchmark, there are a total of 156 texture attributes, and we use all of them for texture concepts (see Appendix for more details). However, we experiment in smaller version for texture concepts such as print, floral, striped, dot, linen, marled, and leopard.

*(3) Color Concepts*. This part of ontology is derived from the ISCC-NBS (Inter-Society Color Council-National Bureau of Standards) color dictionary. An interesting reflection of the validity of this dictionary is given by Miller and Johnson-Laird in 1976. Three kinds of notions are included: hue, brightness, and saturation concepts. There are twenty-eight hue concepts (Table 4) which can be combined with five brightness concepts (very dark, dark, medium, light, and very light) and four saturation concepts (grayish, moderate, strong, and vivid). Certain combinations of brightness and saturation concepts have a perceptual meaning. For instance, the concept “brilliant” is an association of the light and strong concepts. Axioms are contained in the ontology to express those kinds of associations. The mentioned color concepts are especially good for identifying fashion color because the HSV color model is close to human color perception.

In fashion retrieval, it is necessary to check whether the query image has the same color with retrieved ones or not. To solve the problem, color value and color set similarity is recommended to use to compute scores for ranking retrieval results. A special treatment is given to the color attribute, for two reasons: First, the color is described by categorical values (red, blue, yellow, and so on) which have been mentioned in color concepts, but the dissimilarity between two colors can be calculated if the names are mapped into HSV values. Second, the color attribute can take several values for the same item (e.g., a shirt is red and white). In order to compare colors of two cloth items, two concepts need to be introduced: dissimilarity between two colors and dissimilarity between two color images.

Assuming that color *c*_*i*_ is described in the HSV space as (*H*, *S*, *V*), [*H* ∈ 0,360),  *S* ∈ [0,100],  *V* ∈ [0,100], the dissimilarity distance for two values of color is defined in Algorithm 4.

To identify the dissimilarity between two color images, histogram intersection [44] is selected to evaluate the difference between two color distributions of a fashion image. With a given pair of histograms, *H*(*I*) and *H*(*I*′) of images *I* and *I*′, suppose that each one contains *n* bins; then, the histogram intersection *d*(*H*(*I*), *H*(*I*′)) is defined as follows:

(4) $$\begin{array}{c} d\left(H\left(I\right),H\left(I^{\prime }\right)\right)=\frac{\sum_{j=1}^{n}\min\left(H_{j}\left(I\right),H_{j}\left(I^{\prime }\right)\right)}{\sum_{j=1}^{n}H_{j}\left(I^{\prime }\right)}. \end{array}$$

With the dissimilarity between two colors and dissimilarity between two color images, we can reduce the searching space to improve retrieval performance. Histogram intersection is applied in general retrieval tasks (color option is not used).

#### 4.3.2. Specific Fashion Concepts

**Figure 14:**
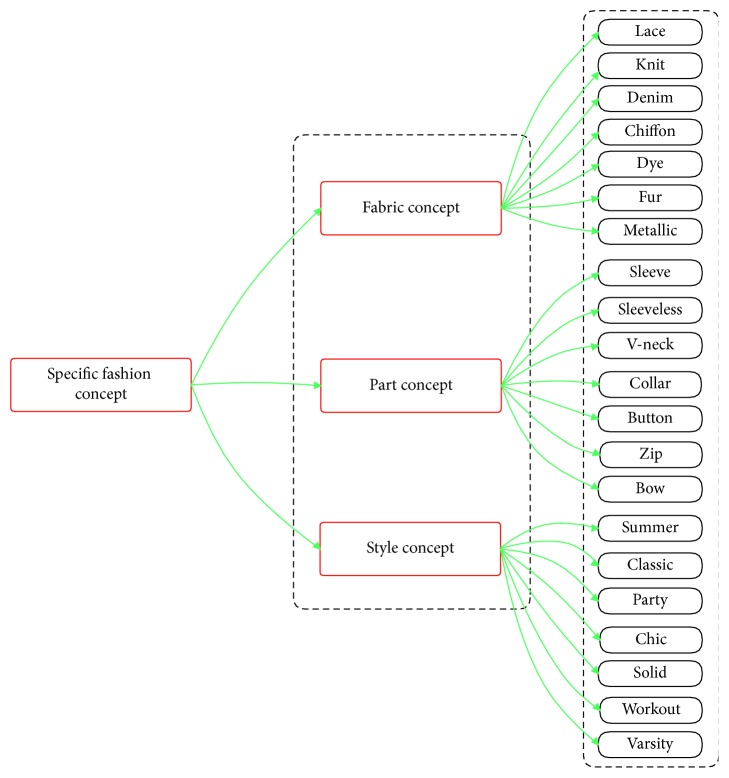
Specific fashion concept ontology.

Specific fashion concepts consisted of fabric concepts, part concepts, and style concepts. These concepts can only appear in clothes, so we call them specific concepts. Thus, we cannot use multitask learning-based models, as mentioned in [37], to solve the attribute prediction problem. Because the specific fashion attributes can be expanded in quantity quickly, multitask learning-based models have to be trained again with a larger dataset whenever a new attribute is added to the system. Local multitask learning is proposed to solve this problem (mentioned in Section 5) (see Figure 14 for specific fashion concepts).

*(1) Fabric Concepts*. This part of ontology has been inspired by results from the DeepFashion dataset (Liu et al. [8]). In category and attribute prediction benchmark, there are a total of 218 fabric attributes, and we use all of them for fabric concepts (see Appendix for more details). However, we experiment in smaller version for fabric concepts such as lace, knit, denim, chiffon, dye, fur, and metallic.

*(2) Part Concepts*. This part of ontology has been inspired by results from the DeepFashion dataset (Liu et al. [8]). In category and attribute prediction benchmark, there are a total of 216 part attributes, and we use all of them for part concepts (see Appendix for more details). However, we experiment in smaller version for part concepts such as sleeve, sleeveless, v-neck, collar, button, zip, and bow.

*(3) Style Concepts*. This part of ontology has been inspired by results from the DeepFashion dataset (Liu et al. [8]). In category and attribute prediction benchmark, there are a total of 230 style attributes, and we use all of them for style concepts (see Appendix for more details). However, we experiment in smaller version for style concepts such as summer, classic, party, chic, solid, workout, and varsity.

## 5. Attribute Learning

To provide fine-grained information to the CFOR system, attribute learning is a most important task which should be optimized in both time-processing performance and ability to deal with large-scale imbalanced datasets.

### 5.1. Framework

**Figure 15:**
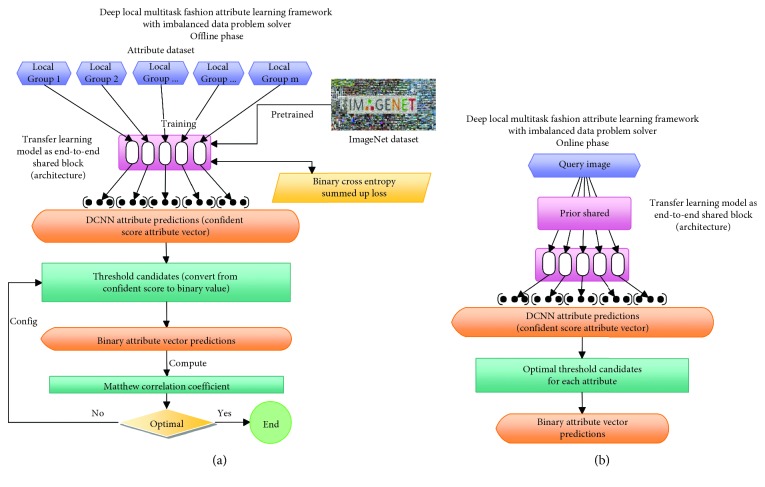
Local MTL with an imbalanced data problem solver framework. (a) Offline phase. (b) Online phase.

As mentioned in Section 1, local multitask learning is considered in attribute learning. The proposed framework (shown in Figure 15 including online and offline phases) has three parts in total. The first part aims to introduce the local multitask transfer learning model with loss function in exploiting attributes' inner-group correlations. The second part shows an imbalanced data solver based on MCC without any revision in the pretrained model as well as loss function. The third part mentions prior knowledge for local attribute grouping to support local MTL.

The input and output of the learning framework will be images and their attribute vectors, respectively. However, with the local grouping role, the attribute vector's size will be based on the number of attributes in each group. The dataset should be merged or split based on the local grouping role.

To evaluate the effectiveness of the proposed framework, we apply it in the fashion field and split the dataset into five local groups: fabric, shape, part, style, and texture. Because fashion has lesser intergroup correlations, the shared block should be designed to optimize the effectiveness of inner-group correlations to improve the overall performance. However, in crowd attributes (such as activities, locations, and participants), intergroup correlations should be taken into account to improve performance. Thus, the shared block should be modified to adapt to the context.

### 5.2. Deep Multitask Learning

Our aim is to estimate a number of fashion attributes via a joint estimation model. However, with the dynamic attributes, MTL which supports creating a joint estimation model becomes vulnerable in the training phase due to its nonusability when the number of attributes increases. Thus, the local grouping method can help solve this situation.

#### 5.2.1. Framework in Detail

In experiments, the proposed framework treats the query image and then outputs 7 attribute scores per group for 5 groups as a confident score vector which is then thresholded to get binary outputs. The architecture is described in detail below.

Figure 15 shows the overall structure of the proposed method. For each group, we suppose a training set with *N* fashion images; each of them has *M* attributes. The dataset is denoted as *D*={*X*, *Y*}, where *X*= {*X*_*i*_}_*i*=1_^*N*^ and *Y*={{*y*_*i*_^*j*^}_*j*=1_^*M*^}_*i*=1_^*N*^, in which will be presented as a one-hot vector of the sample label. Inspired by the study in [25], we use an end-to-end DNN architecture as a shared block to learn joint representations for all tasks. The loss function was binary cross entropy, and activation function used was sigmoid at the output layer to make it simple and easy to change the DNN architecture.

#### 5.2.2. Loss Function

Loss function can be computed as a sum of binary cross entropy loss of all labels (Equation (5)); this is the effective way to handle multitask learning without configuration in the DNN model:

(5) $$\begin{array}{c} ℒ\left(\theta \right)=-\frac{1}{N}\overset{N}{\underset{i=1}{\sum }}\left[y_{i}\;\log\left(\hat{y_{i}}\right)+\left(1-y_{i}\right)\;\log\left(1-\hat{y_{i}}\right)\right]\\ =\;-\frac{1}{N}\overset{N}{\underset{i=1}{\sum }}\overset{M}{\underset{j=1}{\sum }}y_{i}^{j}\;\log\left({\hat{y}}_{i}^{j}\right)+\left(1-y_{i}^{j}\right)\;\log\left(1-{\hat{y}}_{i}^{j}\right), \end{array}$$

where *y*_*i*_ is the multioutput sample label, *y*_*i*_^*j*^ is the sample label for an attribute, *y*_*i*_′ is the multioutput prediction for a sample, ${\hat{y}}_{i}^{j}\in \left(0,1\right):\sum_{j}{\hat{y}}_{i}^{j}=1\forall i,\;j$ is the prediction for a sample in an attribute, *N* stands for the number of samples, and *M* stands for the number of attributes.

#### 5.2.3. Network Architecture

**Figure 16:**
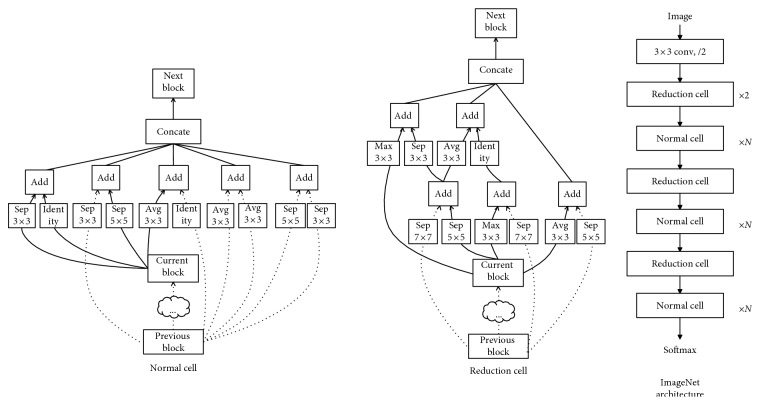
Best normal cells and reduction cells identified with CIFAR-10 and ImageNet architecture (right) are built from the best convolutional cells [36]. Zoph et al. built two types of cells because they want to create architectures for images of any size. While normal cells return a feature map which has the same dimension, reduction cells return a feature map with height and width reduced by a factor of two.

*(1) NASNet*. By producing network architectures automatically, NASNet reconstructs an optimal model by generating architectures on a smaller dataset and expanding it to a larger one. By experiments, they look for the best cells on the CIFAR-10 dataset and then apply them to the ImageNet [33] dataset by stacking together more copies of them, each with their own parameters (Figure 16). The created model was proved to get a 1.2% improvement in top-1 accuracy compared to the best human-invented architectures. As mentioned above, NASNet shows its effectiveness over previous architectures, and it also has a transfer learning model in a large diverse ImageNet dataset [33]. Taking advantage of the NASNet pretrained model on ImageNet, we apply transfer learning in the DeepFashion [8] dataset to speed up convergence and improve performance. When applying NASNet, we also add a dropout layer to reduce overfitting. It is a good consideration to use the NASNet model generation algorithm to make an adaptive model for the DeepFashion dataset. However, NASNet consumes a bunch of time and hardware resources to generate the model and train from scratch. Because of our limitation in hardware, only transfer learning is applied.

*(2) ResNet*. ResNet, a careful human-invented architecture, has been created with the proposed residual blocks. Thanks to them, this architecture has an ability to minimize the effect of the degradation problem when learning deeper and deeper in a complicated deep network. The core idea is to force the network to learn an identity mapping by learning the residual of input and output of some layers (or subnetworks).

Suppose the input of the subnetwork is *x* and the true output is *H*(*x*). Instead of learning a direct mapping of *x* to *y* with a function *H*(*x*) (a few stacks of nonlinear layers) with *x* denoting the inputs to the first of these layers, they define the residual function (assuming that the input *x* and output *H*(*x*) are of the same dimensions) using [35]

(6) $$\begin{array}{c} F\left(x\right)=H\left(x\right)-x. \end{array}$$

As we are interested in finding the true, underlying output of the subnetwork, we then rearrange this original function into *H*(*x*) = *F*(*x*) + *x*, where *x* and *F*(*x*) correspond to the stack of nonlinear layers and the identity function (input = output).

**Figure 17:**
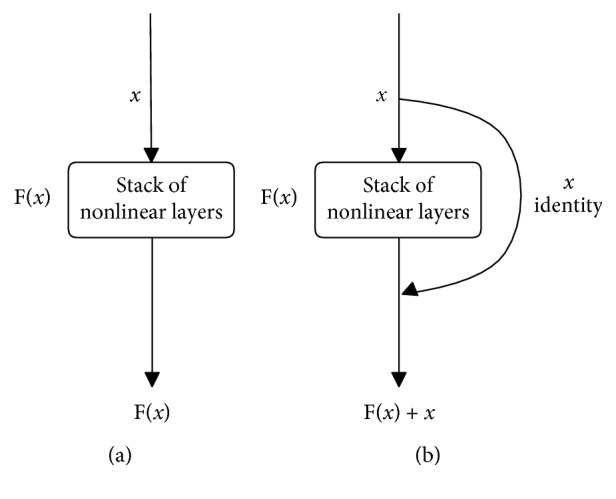
Difference between the original block (right) and the residual block (left) [35].

These things make differences between ResNet and original neural network (plain network) (Figure 17). While the original neural network will learn *H*(*x*) directly, ResNet models the layers to learn the residual of input and output of subnetworks (stack of nonlinear layers). With this innovation, in the classification task on the ISVRC2015, this model has excellently won the first place with a top-5 test error rate of 3.57%. The extremely deep representations also have excellent generalization performance on other recognition tasks: ImageNet detection, ImageNet localization, COCO detection, and COCO segmentation in ILSVRC and COCO 2015 competitions. As mentioned above, ResNet shows its effectiveness over previous architectures, and it also has a transfer learning model in a large diverse ImageNet dataset [33]. Because of that, ResNet also fits well in our requirements.

We will do experiments on ResNet [35] and NASNet [36] architectures to find out which one is suitable for each specific task in our CFOR system. In our fashion retrieval experiments, the category classifier task and region classifier task are applied transfer with single-task learning, while fashion attribute recognition is applied local multitask learning. Besides, to adapt to large-scale datasets and reduce the effect of overfitting, we recommend changing the final fully connected layer to the global average pooling layer along with dropout. These changes are also shown in experiments in Section 7.

#### 5.2.4. Local Multitask Learning for Fashion Attribute

We separate the fashion attribute dataset into five groups: fabric, part, style, shape, and texture. Each group will be applied an individual MTL model. By this, when any new attribute is added, only the group that attribute belongs to is trained again and we can reuse the remaining models. Moreover, inner-group correlations in each group can be learned internally to raise the overall performance.

#### 5.2.5. Imbalanced Data Problem Solving

Thresholds are put after confident score prediction to determine the binary value of each binary attribute. Usually, thresholds are capped at 0.5. However, with imbalanced data, that value is not always the best one, while predicted outputs are often bias to more data classes. By applying MCC in configuring each attribute threshold value, we hope to find the optimal one for solving the imbalanced data problem.

### 5.3. Matthews' Correlation Coefficient

MCC, which is a discriminative version of Pearson correlation in binary variables, has a value between *−*1 and +1. A coefficient of +1 represents a perfect prediction, 0 an average random prediction, and *−*1 an inverse prediction. MCC can help measure the quality of binary classification. Thus, we can base on MCC to change the threshold value which is suitable for each class in the imbalanced dataset.

With two binary variables *x* and *y* showing the presence or absence of an attribute in objects, *t*_*p*_, *t*_*n*_, *f*_*p*_, and *f*_*n*_ are, respectively, the number of true positives, true negatives, false positives, and false negatives, and MCC is defined as [2]

(7) $$\begin{array}{c} \text{MCC}=\text{matthews\_corrcoef}=\frac{\text{cov}\left(x,y\right)}{\sigma_{x}\sigma_{y}}=\;\frac{t_{p}\times t_{n}-f_{p}\times f_{n}}{\sqrt{\left(t_{p}+f_{p}\right)\left(t_{p}+f_{n}\right)\left(t_{n}+f_{p}\right)\left(t_{n}+f_{n}\right)}}. \end{array}$$

In Equation (7), if any of the four sums in the denominator is zero, the denominator can be arbitrarily set to one, and this results in Matthews' correlation coefficient being masked as zero, which can be shown to be the correct limiting value.

With a given threshold between 0 and 1, MCC can base on the predicted output and images label to give out a score. The higher the score, the better the classification quality and the more optimal the threshold in attribute prediction. After testing a sufficiently large number of thresholds, we can find out the best one for each attribute that minimizes the impact of imbalanced data.

MCC can be called an application of phi correlation coefficient (*ϕ*)—a binary version of the Pearson correlation coefficient (PCC) with 2 binary variables *x* and *y* which show the presence or absence of an attribute in objects. The Pearson correlation coefficient (also called the correlation coefficient in short) is a bivariate correlation which is a measure of the linear correlation between two variables *x* and *y*. It has a value between +1 and −1, where +1 is total positive linear correlation, 0 is no linear correlation, and −1 is total negative linear correlation.

Let PCC be

(8) $$\begin{array}{c} {\text{PCC}}_{x,y}=\frac{\text{cov}\left(x,y\right)}{\sigma_{x}\sigma_{y}}=\frac{E\left[xy\right]-E\left[x\right]E\left[y\right]}{\sqrt{Var\left[x\right]\;*\;Var\left[y\right]}}=\frac{n_{11}n-n_{1•}n_{•1}}{\sqrt{n_{0•}n_{1•}n_{•0}n_{•1}}}, \end{array}$$

where

(9) $$\begin{array}{c} E\left[x\right]=\frac{n_{1•}}{n},\\ \text{Var}\left[x\right]=\frac{n_{0•}n_{1•}}{n},\\ E\left[y\right]=\frac{n_{•1}}{n},\\ \text{Var}\left[y\right]=\frac{n_{•0}n_{•1}}{n},\\ E\left[xy\right]=\frac{n_{1•}n_{•1}}{n^{2}}, \end{array}$$

cov(*x*, *y*) is the covariance of two variables *x* and *y*, *σ*_*x*_ is the standard deviation of variable *x*, *σ*_*y*_ is the standard deviation of variable *y*, and *n* is the total number of observations. Two binary variables are considered positively associated if data fall along the diagonal cells and are considered negatively associated if they fall off the diagonal. Let us consider a 2 × 2 status table for two binary variables *x* and *y* (Table 5).

Here, *n*_11_,  *n*_10_,  *n*_01_,  and  *n*_00_ are nonnegative counts of numbers of observations that sum to *n*. *n*_1•_, *n*_0•_, *n*_•1_,  and  *n*_•0_ are total counts of numbers of observations when *x*=1,  *x*=0,  *y*=1,  and  *y*=0, respectively.

When *x* and *y* are binary variables,

(10) $$\begin{array}{c} \left(\text{Equation }5\right)⟶E\left[xy\right]=\frac{n_{1•}n_{•1}}{n^{2}}=\;\left(\frac{n_{11}}{n}\;*\;1\;*\;1+\frac{n_{10}}{n}\;*\;1\;*\;0+\frac{n_{01}}{n}\;*\;0\;*\;1+\frac{n_{00}}{n}\;*\;0\;*\;0\right)=\frac{n_{11}}{n}\\ ⟶n_{11}n-n_{1•}n_{•1}=\;n_{11}\left(n_{01}+\;n_{10}+\;n_{11}+\;n_{00}\right)-\left(n_{11}+\;n_{10}\right)\left(n_{11}+\;n_{01}\right)=n_{11}n_{00}-n_{10}n_{01}\;\left(\text{Equation }7\right),\\ \left(\text{Equation }4\right)\left(\text{Equation }6\right)⟶t_{p}=\;n_{11};\;t_{n}=\;n_{00};\;f_{p}=\;n_{10};\;f_{n}=\;n_{01}\left(\text{Equation }8\right),\\ \left(\text{Equation }7\right)⟶PCC_{x,y}=\text{ MCC}. \end{array}$$

When we have multilabels, to find the best thresholds for all of them, we should consider Algorithm 5.

With best threshold for each label, we can use them to get a prediction binary value with minimal effects of the imbalanced data problem.  Algorithm 6 can convert model prediction values to binary values.

### 5.4. Local Attribute Grouping Method

Our grouping method is based on characteristics of general attributes and fashion ones. Thus, we separate attributes into two large groups: a general one and a fashion one. In each group, we define some concept; each will be applied MTL. For the general group, we propose visual concepts which can appear in any kind of object not restricted by fashion, including color, shape, and texture. For the fashion group, we propose concepts that only appear in fashion objects including fabric, part, and style (see Table 6). The grouping method for all experiment attributes is defined by making use of ontology (especially fashion fine-grained concept ontology) in Section 4.

## 6. Searching and Indexing Method in the CFOR System

To make our retrieval system fit for application in the large-scale dataset, indexes for the CFOR system are created to support nonexhaustive similarity search with GPU. To make this work, we inherit the searching algorithm introduced by Johnson et al. (billion-scale similarity search with GPUs [17]) and apply it on the retrieval task in the CFOR system. In searching, the CFOR system helps reduce the searching space by additional information (regions, categories, and attributes) which makes searching more accurate. In indexing, object ontology helps create multi-indexing files so as to decrease searching time. We are concerned with similarity search in vector collections by applying L2 distance in the *k*-selection algorithm.

As far as we know, searching can be separated into exact search (exhaustive search) and compressed search (greedy nonexhaustive search). Let us have [*x*_*j*_]_*j*=0:*n*_(*x*_*j*_ ∈ *ℝ*^*d*^), the given collection of query vectors, and [*y*_*i*_]_*i*=0:*l*_(*y*_*i*_ ∈ *ℝ*^*d*^), the corresponding given image vector database.

### 6.1. Exact Search

Almost all searching algorithms in this type try to compute the full pairwise distance between the query and each data point in the database sequentially or using the index file. To achieve this, we compute the full pairwise distance matrix *D*=[‖*x*_*j*_ − *y*_*i*_‖_2_^2^]_*j*=0:*n*_*q*_, *i*=0:*l*_ ∈ *R*^*n*_*q*_×*l*^. Exact search can help minimize the error in computing distance between the query and each element in the database. However, it takes long time to finish computation because of its exhaustive searching ability which does not suit large-scale searching.

### 6.2. Compressed-Domain Search

Almost all searching algorithms in this type try to compute distance between the query and each data point in the database by applying space transformation, encoding, subspace splitting, or hashing. These methods can help improve searching time by using index files, but they have a trade-off in searching accuracy.

In this method, to take advantage of the power of encoding and approximate computing in searching to raise the retrieval speed, we focus on approximate nearest-neighbour search. In the IVFADC (an inverted index file system with asymmetric distance computing in encoding) indexing structure proposed in [17], encoded database vectors and quantization extraction define the index file. IVFADC distance (*L*_IVFADC_) is computed as the distance between the unencoded query and each encoded database vector in the transformed compressed domain. When *y* is a database vector, we quantize it as

(11) $$\begin{array}{c} y\approx q_{1}\left(y\right)+q_{2}\left(y-q_{1}\left(y\right)\right), \end{array}$$

where *q*_1_ : *R*^*d*^⟶*C*_1_ ⊂ *R*^*d*^ is a coarse quantizer and *q*_2_ : *R*^*d*^⟶*C*_2_ ⊂ *R*^*d*^ is a fine quantizer. As the sets are finite, *y* can be reconstructed by the index of the coarse quantizer and that of the fine quantizer. Because *y* has been encoded, to compute the distance between a query vector *x* and a vector *y* in the database, we need an approximate searching distance, as proposed by Faiss [17]; asymmetric distance computation (ADC) which helps compute distance between an unencoded input query and encoded vectors in the database is applied:

(12) $$\begin{array}{c} L_{\text{ADC}}=k-\underset{i=0:l}{\arg\min}{\left\|x-q\left(y_{i}\right)\right\|}_{2}, \end{array}$$

where *L*_ADC_ is the computed distance and *k* is the number of nearest neighbours of *x*.

While the search is not exhaustive, vectors for which the distance is computed are then selected based on the first-level quantizer *q*_1_. Our searching method distance is then needed to adapt to compressed domain and help find out the distance between the query and each coarse-level centroid. The following equation shows the compressed-domain-transformed distance:

(13) $$\begin{array}{c} L_{\text{IVF}}=\tau -\underset{c\in C_{1}}{\arg\min}{\left\|x-C\right\|}_{2}, \end{array}$$

where *L*_IVF_  is the transformed distance in the compressed domain and *τ* is the multiprobe parameter—the number of coarse-level centroids we have.

The quantizer operates a nearest neighbour search with exact distances. Thus, we need to combine two mentioned distances to make the searching method visible. The IVFADC search is then established and can be computed as *L*_IVFADC_ (distance between the unencoded query and each encoded database vector in the transformed compressed domain):

(14) $$\begin{array}{c} L_{\text{IVFADC}}=\underset{i=0:l\;\;s.t.q_{1}\left(y_{1}\right)\in L_{\text{IVF}}}{k-\arg\min}{\left\|x-q\left(y_{i}\right)\right\|}_{2}. \end{array}$$

Hence, IVFADC not only is based on the same distance estimations as the coarse-fine quantization but also can be computed as a subset of vectors.

Finally, the inverted indexing file, the corresponding data structure, groups the vectors *y*_*i*_ into |*C*_1_| inverted lists *I*_1_,   …,  *I*_|*C*_1_|_ with homogeneous *q*_1_(*y*_1_).

To meet the requirement of our targets in searching large-scale data, compressed-domain search is then applied in the CFOR system for image retrieval.

## 7. Results and Discussion

To evaluate the effectiveness of the CFOR system, our experiments are implemented on a specific dataset with many different tasks supporting retrieval including category classification, attribute prediction, and similarity ranking retrieval.

In category classification, we prove the effectiveness of two architectures: NASNet and ResNet, to find out which is suitable for classification in the CFOR system.

In attribute prediction, we prove the usefulness of our proposed local multitask learning framework with our suggested imbalanced data solver in both NASNet and ResNet. Our experiments set up the local MTL framework with the following attribute tasks: local multitask, multitask, and single-task prediction with and without applying MCC. Threshold modification for output prediction can reduce training time by taking advantage of transfer learning, minimize parameter quantity, and simplify loss function. Compared with data augmentation, local MTL does not increase overfitting.

Our experiments are conducted using Python on computers with the following specifications: Intel Xeon E5-2650 v2 16-Core Processor 2.6 GHz 8.0 GT/s 20 MB, Ubuntu operating system 16.04 64-bit, 196 GB RAM, Nvidia 1080Ti GPU 12 GB RAM.

### 7.1. Data

**Figure 18:**
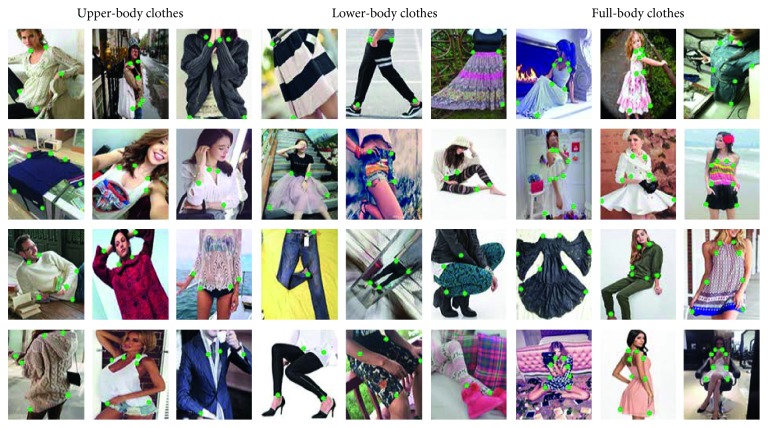
Images from the DeepFashion dataset obtained from different views and complicated background.

**Figure 19:**
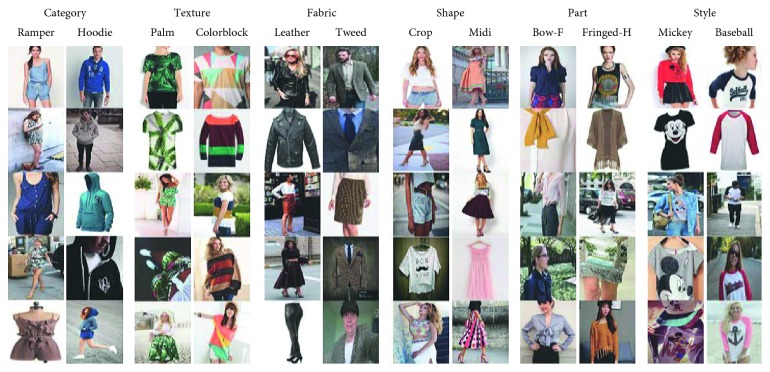
Images from the DeepFashion dataset annotated with different labels based on details of input of the current model concern.

Our fashion retrieval system was built on a subset of approximately 300,000 images of DeepFashion. In the DeepFashion dataset, objects from different aspects are caught in complicated background. The input image in the dataset is annotated with different labels based on details (fine-grained) of input of the current model concern, i.e., rich annotation. The samples given in Figures 18 and 19 show more details about the DeepFashion dataset.

In testing, we employ part of the benchmark data to fine tune the trained models. We ensure that there are no fashion item overlaps between fine-tuning and testing sets. The dataset includes ∼220,000 images of the training set, 40,000 images of the validating set, and 40,000 images of the testing set split by authors in [35]. However, in attribute learning, we limited the number of attribute labels used for testing and the number of training images for specific attributes to make an imbalanced attribute dataset (IAD-35) so as to prove our proposed methods. Tables 7 and 8 show the imbalanced data problem in the IAD-35 dataset in both local grouping- and nonlocal grouping-applied situations (we consider two attributes belong to fewer data attribute groups or more data attribute groups if the ratio in samples between them is higher than 3). These tables show a big difference in the imbalance on the dataset when comparing the global case (without grouping) and the local grouping case.

These tables show that the number of attributes that have more data is increased in quantity when applying the local grouping method. This will help reduce the imbalanced data problem when training model in each local group. If we keep training in the whole imbalanced dataset, the imbalanced gap between attributes becomes higher which makes the training model easily biased.

### 7.2. Testing and Competing Method

To compare the results with other research works easier, we use top-*k* accuracy for category classification and top-1 recall for attribute multitask learning. To be clear, we define these comparison methods in general:

For *single-task classification*, let  *T* be a dataset consisting of single-label examples (*x*_*i*_, *y*_*i*_), 1 ≤ *i* ≤ *n*,  (*x*_*i*_ *ϵ* *X*,  *y*_*i*_ ∈ *Y*), where *Y* is a group of possible classes. Let *h* be the classifier and *Z*_*i*_=*h*(*x*_*i*_) be the set of labels predicted by *h* for the corresponding example *x*_*i*_.

To obtain top-*k* accuracy, check whether the target label is one of the top-*k* predictions (the *k* ones with the highest probabilities). The top score is computed as the times a predicted label matched the target label, divided by the number of examples evaluated:

(15) $$\begin{array}{c} \text{top}‐k\text{ accuracy}=\;\frac{1}{n}\overset{n}{\underset{i=1}{\sum }}\left|Z_{i}{\;\cap }^{}y_{i}\right|. \end{array}$$

For *multitask learning with binary label*, let *T* be a dataset consisting of *n* multilabel examples (*x*_*i*_, *Y*_*i*_), 1 ≤ *i* ≤ *n*,  (*x*_*i*_ *ϵ* *X*,  *Y*_*i*_ ∈ *Y*= {0,1}^*c*^), where *c* is the amount of label. Let *h* be the multilabel classifier and *Z*_*i*_=*h*(*x*_*i*_) be the set of label memberships predicted by *h* for the example *x*_*i*_.

Accuracy, for each instance, is defined as the proportion of the predicted correct labels to the total number (predicted and actual) of labels for that instance. Overall accuracy is the average across all instances:

(16) $$\begin{array}{c} \text{accuracy}=\;\frac{1}{n}\overset{n}{\underset{i=1}{\sum }}\frac{\left|Z_{i}{\;\cap }^{}Y_{i}\right|}{\left|Z_{i}{\;\cup }^{}Y_{i}\right|}. \end{array}$$

Precision is a proportion of predicted correct labels to the total number of actual labels, averaged over all instances:

(17) $$\begin{array}{c} \text{precision}=\;\frac{1}{n}\overset{n}{\underset{i=1}{\sum }}\frac{\left|Z_{i}{\;\cap }^{}Y_{i}\right|}{\left|Z_{i}\right|}. \end{array}$$

Recall is a proportion of predicted correct labels to the total number of predicted labels, averaged over all instances:

(18) $$\begin{array}{c} \text{recall}=\;\frac{1}{n}\overset{n}{\underset{i=1}{\sum }}\frac{\left|Z_{i}{\;\cap }^{}Y_{i}\right|}{\left|Y_{i}\right|}. \end{array}$$

Mean average precision (MAP), which provides a single-figure measure consisting of precision and recall, is used to evaluate the effectiveness of retrieval results. It evaluates the extent to which the correct retrieval results are in the high rankings. Among evaluation measures, especially MAP has been shown to have good discrimination and stability. For a single information need, average precision (AP) is the average of the precision value obtained for the set of top-*k* images existing after each relevant image is retrieved:

(19) $$\begin{array}{c} \text{average precision AP}\left(n,R_{j}\left(q_{j}\right)\right)=\;\frac{\sum_{k=1}^{n}P\left(k,R_{j}\left(q_{j}\right)\right)\times \text{rel}\left(k\right)}{\text{number of relevant images}},\\ \text{mean average precision MAP}\left(Q\right)=\;\frac{1}{\left|Q\right|}\overset{\left|Q\right|}{\underset{j=1}{\sum }}AP\left(n,R_{j}\left(q_{j}\right)\right), \end{array}$$

where *q*_*j*_ ∈ *Q* in which *q*_*j*_ is a query image in the *Q* query set; *R*_*j*_(*q*_*j*_) is a set of ranked retrieval images for query *q*_*j*_; *P*(*k*) is the precision at *k*; and rel(*k*) is an indicator function equaling 1 if the image at rank *k* is relevant, or else zero.

To be explicit, we divide the experiment process into an academic one and an application one.

### 7.3. Results and Discussion

In the CFOR system, object ontology is useful in controlling training flow which impacts the performance of object category classification and attribute multitask classification. For object category classification, ontology controls the amount of training data through concepts. For attribute multitask classification, ontology manages local grouping which directly affects the performance of the proposed local imbalanced data solver on the large-scale dataset.

In this section, we will evaluate the effectiveness of different deep networks with the support of ontology on both category classification and attribute multitask classification in the CFOR system to pick out the best architecture for training the system. We will also compare our results with FashionNet [8].

#### 7.3.1. Category Classification

**Figure 20:**
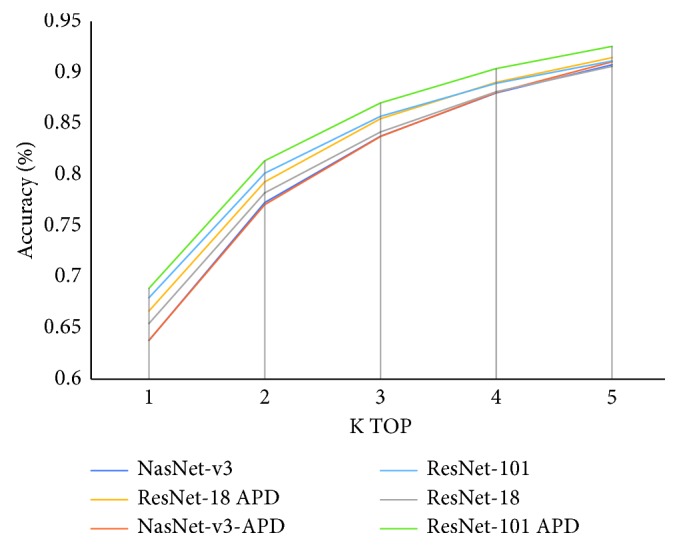
Accuracy plot for top-*k* accuracy in category classification.

We compare the performance between different deep architectures including NASNet, ResNet-18, ResNet-101, FashionNet, NASNet with average pooling dropout (NASNet APD) (proposed by us), and ResNet with average pooling dropout (ResNet APD) (proposed by us). These experiments will be evaluated by top-*k* accuracy (Table 9 and Figure 20). Our target is to find out the best possible architecture to apply as a core network of the CFOR system. This step can be mentioned as a preparation step before applying the CFOR system for fashion retrieval.

**Figure 21:**
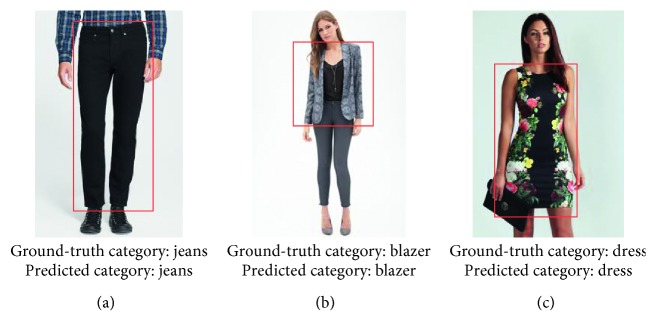
An example of the category prediction results of best object category classification models in the CFOR system.

The result of category classification by ResNet-18 APD is higher than 1.23% (at *k* = 1) after removing nodes and making average pooling in the ResNet-18 architecture (compared with the original ResNet-18 architecture). This increased value is 0.93% with the ResNet-101 architecture (compared with the original ResNet-101 architecture) and 0.02% with the NASNet v3 architecture (compared with the original NASNet v3 architecture). The ResNet-101 APD architecture (the best architecture addressed) outperformed the FashionNet architecture (the best performing architecture in category classification on the DeepFashion dataset versus others such as WTBI or DARN), and the value is 4.6% with *k* = 3 and 2.58% with *k* = 5 (see Figure 21 for some example results of the best object category classification model) [45].

Based on the above experimental results, the ResNet-101 architecture provides better classification and higher performance compared to others (NASNet and ResNet-18). For this reason, we propose ResNet-101 as the core network architecture for training classification models.

#### 7.3.2. Attribute Learning

Attribute multitask learning is an important part of the CFOR system. In this section, we evaluate the performance of the proposed local imbalanced data solver with MCC in dealing with the imbalanced attribute data on the large-scale fashion dataset.

Precision is the proportion of relevant instances among the retrieved instances which consider both true positives and false positives in each attribute. However, the number of true positives and false positives is bias because of the imbalanced data problem. Thus, precision can also be affected by the imbalanced data problem. Otherwise, recall, which cares about true-positive labels but not false-positive labels, will be used to evaluate experiments because of its good reflection for fewer data attributes:

(20) $$\begin{array}{c} \text{precision}=\;\frac{t_{p}}{t_{p}+f_{p}},\\ \text{recall}=\;\frac{t_{p}}{t_{p}+f_{n}}. \end{array}$$

In the *first experiment*, we show the effectiveness of local MTL over STL and MTL in fashion attributes (Table 10). In STL and MTL, we apply the dataset arranged as in Table 7, while in local MTL, we apply the dataset arranged as in Table 8, which has been split into 5 smaller local groups.

**Figure 22:**
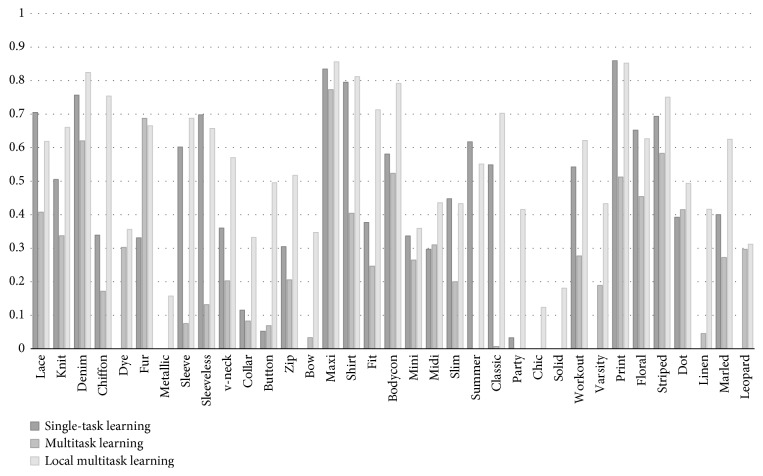
Recall graph of 14 attributes in STL and local MTL.

Local MTL gets over STL and MTL in 28/35 attributes with a 54.70% recall rate (higher than that in STL (17.06%) and that in MTL (28.70%)). While a single task shows its weakness in fewer data attributes and multitasks get struggled with the serious imbalanced problem and lesser intergroup correlations in fashion data, local MTL can lower their negative influences as well as widen the positive effect of inner-group correlations on attribute learning. Thus, local MTL gets over STL and MTL in 13/15 fewer sample attributes (Figure 22).

In the *second experiment*, we show the effectiveness of MCC in solving the imbalanced data problem (Table 11). Also, in this experiment, local MTL is applied with the dataset arranged as in Table 8, which has been split into 5 smaller local groups.

**Figure 23:**
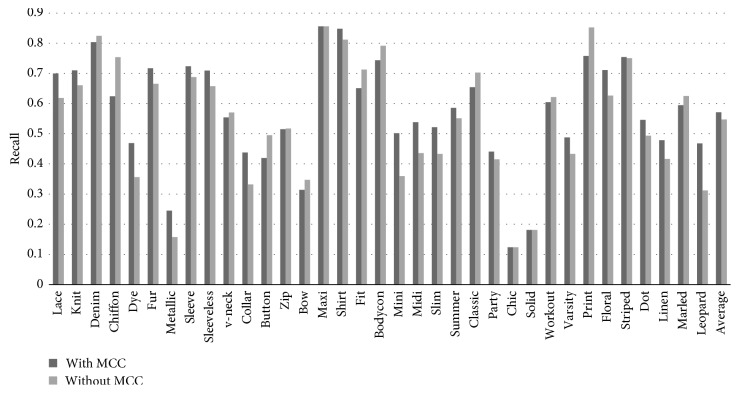
Recall graph of 35 attributes using local multitask models with and without MCC.

Based on the experiment, comparison of chic, solid, and maxi attributes which have equal accuracy between MTL with and without MCC shows that MTL with MCC had higher recall compared to that without MCC in 20/35 remaining attributes. The overall performance increases about 3%. For attributes with fewer data, MTL with MCC had higher recall compared to that without MCC in 9/14 attributes. The overall performance for these fewer data attributes increases 5.14% (see Figure 23 for more details).

**Figure 24:**
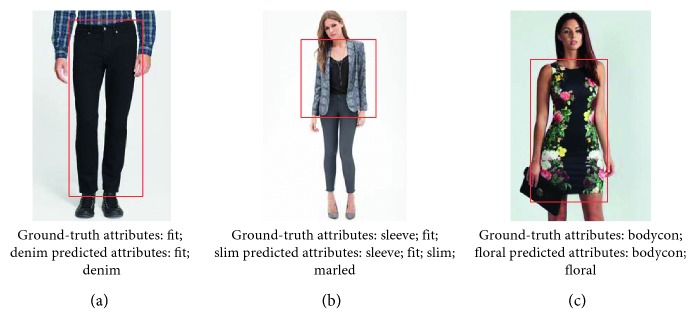
An example of the attribute prediction results of best object attribute classification models in the CFOR system.

Also, in Figure 24, some example results of the attribute multitask classification model are visualized for proving the effectiveness of our proposed method.

### 7.4. Retrieval in CFOR System

**Figure 25:**
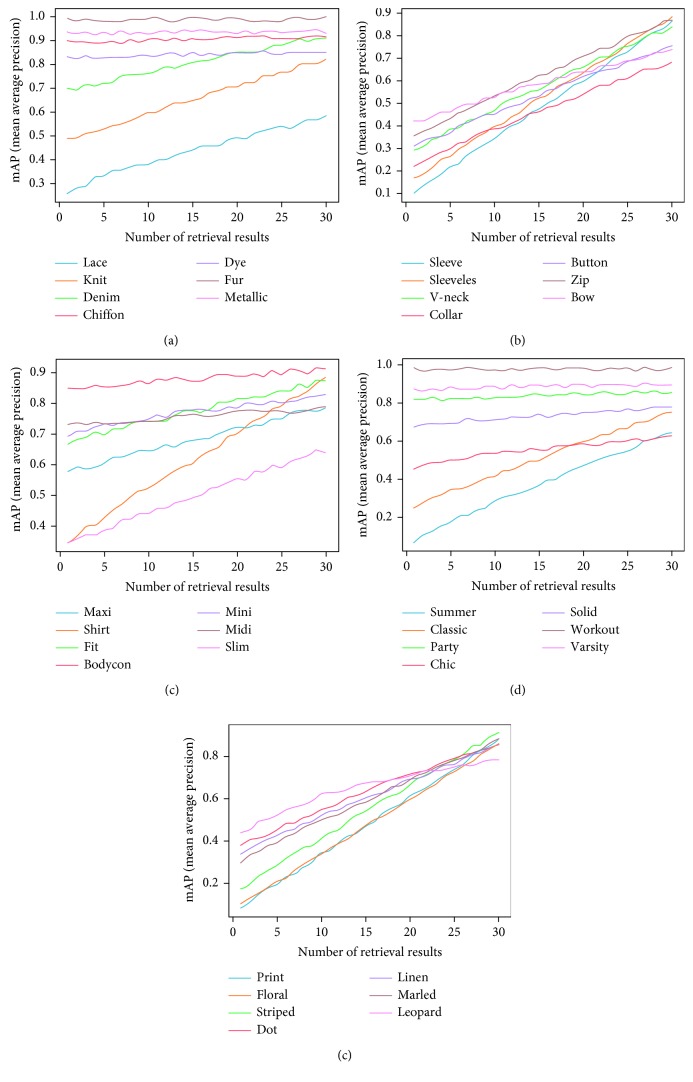
MAP graph of 35 attributes from MAP@1 to MAP@35 in similarity retrieval evaluation for (a) fabric, (b) part, (c) shape, (d) style, and (e) texture groups.

**Figure 26:**
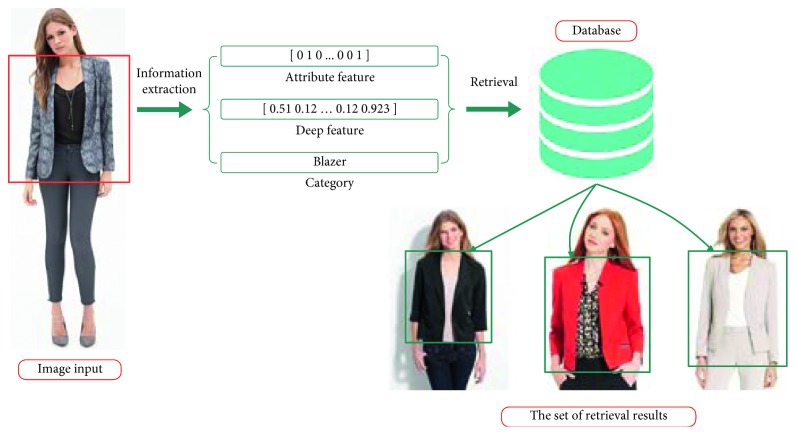
An example of the retrieval results of the CFOR system.

In this experiment, we test the retrieval ability of the CFOR system by using MAP from 1 retrieval result for each query (MAP@1) to 30 retrieval results for each query (MAP@30) so as to evaluate the effectiveness. The similarity retrieval experiment will check whether the extracted attributes in retrieved images are matched with ground-truth attributes in query image. The retrieval method will be based on deep features and over 35 attributes. After experimenting in 35 attributes belonging to 5 groups, the starting MAP@5 is acceptable (hovering 0.531) which shows the effectiveness of the searching method. The MAP@30 hovers 0.815, and the trend keeps rising which shows consistency and stabilization of information prediction methods in the CFOR system (Figure 25). A simple visualization of the retrieval process in the CFOR system is shown in Figure 26.

Besides, to clarify the potential of the CFOR system in real-world applications, Table 12 demonstrates times needed for training, testing, and updating the system.

## 8. Conclusion and Perspective

This work presents the coarse-to-fine object retrieval system, a learning framework for e-commerce online retrieval, which is supported to deal with large-scale imbalanced datasets. The framework can impact input and output as well as reconstruct datasets from the coarse-grained level to the fine-grained level and is believed to be an effective method in improving learning performance designed for retrieval. For input reconstruction, the framework based on ontology is used for threading training flow, local grouping in multitask attribute learning, and hierarchical storage and retrieval. For output optimization, we take advantage of MCC to minimize the effect of the imbalanced dataset on multitask attribute learning.

Through extensive experiments, we demonstrate the applicability of object ontology in improving training flow, the effectiveness of different deep networks (ResNet and NASNet) applied on important tasks in fine-grained retrieval, and the usefulness of local multitask attribute learning and an MCC-based imbalanced data solver in attribute multitask learning. The CFOR system is designed to have flexibility so that it can be optimized easily in the future.

## Figures and Tables

**Algorithm 1:**
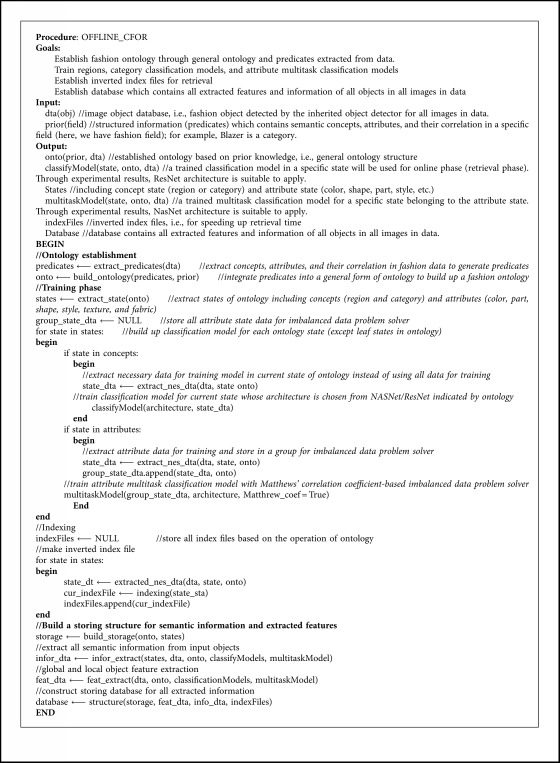
Offline-phase CFOR system algorithm applied in the fashion field.

**Algorithm 2:**
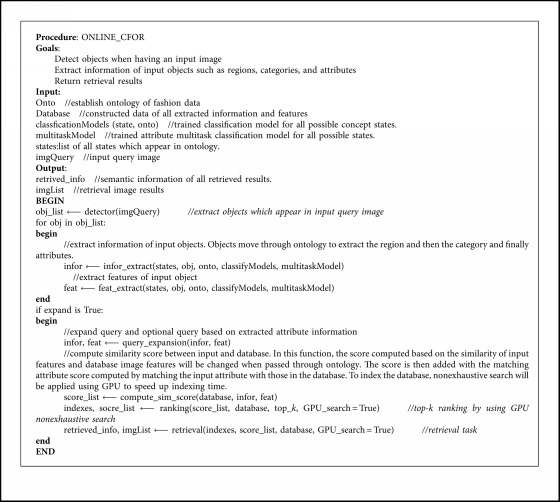
Online-phase (retrieval-phase) CFOR system algorithm applied in the fashion field.

**Algorithm 3:**
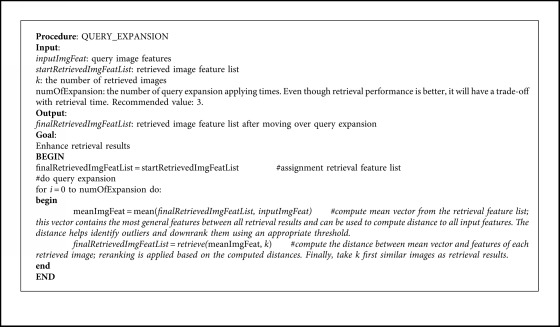
Query expansion for image retrieval.

**Algorithm 4:**
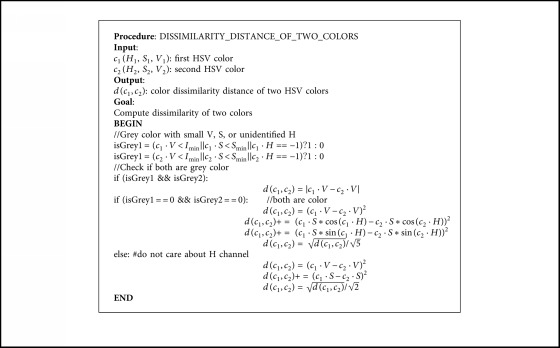
Computation of dissimilarity distance of two colors.

**Algorithm 5:**
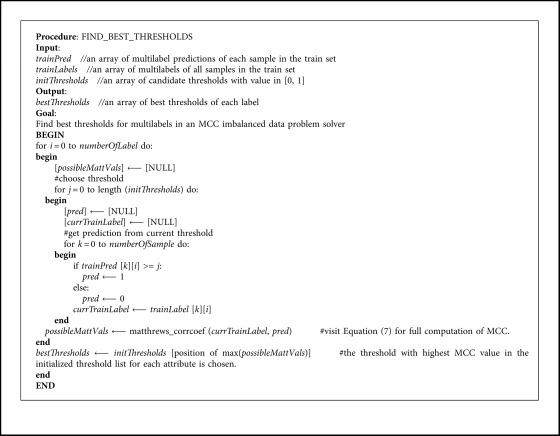
Finding best thresholds for multilabels.

**Algorithm 6:**
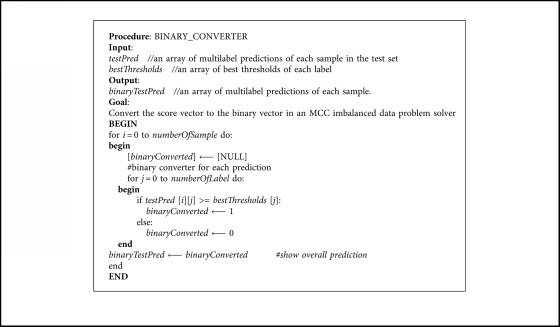
Conversion of the score vector to the binary vector.

**Table 1:** Contributions of CFOR in the offline phase and its comparison with DeepFashion [8].

Criteria	Object ontology (categories/attributes)	Deep learning model	Imbalanced data problem solver	Updating system	Searching method
CFOR system	Object ontology can be implemented on arbitrary objects with flexible modifications. It is not just for fashion. Ontology is a semantic hierarchical tree. It consisted of three levels: (i) Region level (ii) Category level (iii) Attribute level Region level consisted of some parts of objects; each part is linked to the category level. Category level consisted of some fine-grained groups; each member of the group is linked to the attribute level. Attribute level consisted of two coarse-grained groups: visual concept (color, shape, and texture) and specific attribute concept (fabric, part, and style). It is easily implemented on another kind of object with flexible modifications. It is not just for fashion.	ResNet-101 is used in category classification NASNet v3 is used in attribute classification	Based on Matthews' correlation coefficient	Based on transfer learning. Trains smaller models based on ontology and updates the system in a flexible manner in each model	GPU-based nonexhaustive similarity search

FashionNet [8]	The authors do not use the terminology “ontology.” Dataset is organized based on a hierarchical tree. It is implemented just for fashion. It consisted of two-level trees: (i) The first level consisted of category groups (50 categories) (ii) The second level consisted of 5 attribute groups (texture, fabric, shape, part, and style). It does not have color attribute (It treats concepts and attributes as independent tasks of classification).	VGG	Not considered yet	Trains the entire classification model again	Exhaustive search

**Table 2:** Contributions of CFOR in the online phase and its comparison with DeepFashion [8].

Criteria	Query	Retrieval process	Indexing method	Retrieval results
CFOR system	Image + optional semantic information (categories and attributes) extracted automatically from an image	Conducted by deep networks based on object ontology at three levels: region, category, and attribute levels	Quantized inverted indexing is operated by object ontology	Object ontology supports achieving retrieval results. Retrieval results are based on deep global features and attribute vector. Query expansion is used to improve the performance of the retrieval system.

FashionNet [8]	Image	Conducted by deep networks and landmark points (also built up by deep networks)	Inverted indexing	Result obtained is similarity retrieval only.

**Table 3:** Summary of the contribution of each method for each criterion.

Methods	Attribute learning model based on deep features with SVM classifiers (Figure 1)	Attribute learning model based on adaptive attribute domain with independent deep neural networks (Figure 2)	Attribute learning model based on the end-to-end deep neural network as a shared block with adaptive loss functions (Figure 3)	Our proposed deep local multitask learning
Inner-group correlation	Not focused	Focused	Focused	Focused
Intergroup correlation	Not focused	Focused	Focused	Not focused
Imbalanced data solving	Not focused	Not focused	Focused	Focused
Transfer learning	Feedforward only	Applied in each individual network	Not focused but can be applied	Focused
Large-scale data adaption	Not focused	Limited	Focused	Focused
Ontology	Not focused	Not focused but can be applied	Not focused but can be applied	Focused

**Table 4:** Set of hue concepts.

Red	Purple
Reddish orange	Reddish purple
Orange	Purplish red
Orange yellow	Purplish pink
Yellow	Pink
Greenish yellow	Yellowish pink
Yellow green	Brownish pink
Yellowish green	Brownish orange
Green	Reddish brown
Bluish green	Brown
Greenish blue	Yellowish brown
Blue	Olive brown
Purplish blue	Olive
Violet	Olive green

**Table 5:** 

	*y*=1	*y*=0	Total
*x*=1	*n*_11_	*n*_10_	*n*_1•_
*x*=0	*n*_01_	*n*_00_	*n*_0•_
Total	*n*_•1_	*n*_•0_	*n*

**Table 6:** Attribute grouping table.

Group	Concept	Attributes
General	Color	red, orange, yellow, green, blue, etc. (28 × 4 × 5 attributes based on HSV)
	Shape	skinny, fit, bodycon, maxi, etc. (180 attributes)
	Texture	floral, stripe, dot, print, graphic, etc. (156 attributes)

Fashion	Fabric	fur, leather, denim, cotton, etc. (218 attributes)
	Part	sleeve, racerback, hooded, etc. (216 attributes)
	Style	summer, workout, party, etc. (230 attributes)

**Table 7:** IAD-35 for nonlocal grouping in attribute multitask learning.

Attribute	Number of positive samples
**print**	37367
**floral**	24188
**lace**	20434
knit	18498
sleeve	17828
maxi	15990
shirt	14920
denim	13178
striped	11771
chiffon	11735
*sleeveless*	7987
*summer*	7616
*fit*	7489
*classic*	7184
bodycon	6419
mini	6065
v-neck	5493
collar	5458
*button*	5057
*midi*	4660
*dot*	3810
*slim*	3495
*zip*	3266
*linen*	3051
*party*	2882
*marled*	2724
*dye*	2490
*chic*	2099
*fur*	2061
*metallic*	2044
*leopard*	1832
*solid*	1718
*bow*	1669
*workout*	1275
*varsity*	1101

Bold: attributes with more data. Italics: attributes with fewer data.

**Table 8:** IAD-35 for local grouping in attribute multitask learning.

Attribute group	Attribute	Number of positive samples
**Fabric**	**lace**	20434
	**knit**	18498
	denim	13178
	chiffon	11735
	*dye*	2490
	*fur*	2061
	*metallic*	2044

**Part**	**sleeve**	17828
	sleeveless	7987
	v-neck	5493
	collar	5458
	button	5057
	*zip*	3266
	*bow*	1669

**Shape**	**maxi**	15990
	**shirt**	14920
	fit	7489
	bodycon	6419
	mini	6065
	*midi*	4660
	*slim*	3495

**Style**	**summer**	7616
	**classic**	7184
	party	2882
	chic	2099
	*solid*	1718
	*workout*	1275
	*varsity*	1101

**Texture**	**print**	37367
	**floral**	24188
	striped	11771
	dot	3810
	linen	3051
	*marled*	2724
	*leopard*	1832

Bold: attributes with more data. Italics: attributes with fewer data.

**Table 9:** Top-*k* accuracy table between different deep architectures in category classification.

Model	Top-*k* accuracy				
	1	2	3	4	5
FashionNet [8]	—	—	0.8258	—	0.9017
NASNet v3 [20]	0.6382	0.7739	0.8391	0.8817	0.9094
NASNet v3 APD	0.6384	0.7718	0.8388	0.8822	0.9123
ResNet-18 [19]	0.6549	0.7834	0.8433	0.8829	0.9078
ResNet-18 APD	0.6672	0.7942	0.8563	0.8922	0.9164
ResNet-101 [19]	0.6802	0.8027	0.8587	0.8912	0.9132
ResNet-101 APD	0.6895	0.8150	0.87188	0.9057	0.9275

**Table 10:** Recall in STL and MTL for fashion attributes.

Attribute	STL	MTL	Local MTL
lace	0.7049	0.4076	0.6185
knit	0.5051	0.3371	0.6606
denim	0.7567	0.6203	0.8244
chiffon	0.3390	0.1717	0.7538
*dye*	0.0	0.3027	0.3561
*fur*	0.3308	0.6875	0.6654
*metallic*	0.0	0.0	0.1573
sleeve	0.6018	0.0753	0.6876
sleeveless	0.6977	0.1315	0.6574
v-neck	0.3602	0.2025	0.5702
*collar*	0.1152	0.0827	0.3320
*button*	0.0524	0.0690	0.4952
*zip*	0.3048	0.2055	0.5173
bow	0.0	0.0331	0.3471
maxi	0.8345	0.7730	0.8560
shirt	0.7950	0.4042	0.8117
fit	0.3768	0.2464	0.7127
bodycon	0.5808	0.5234	0.7916
*mini*	0.3365	0.2647	0.3593
*midi*	0.2969	0.3099	0.4356
*slim*	0.4474	0.2	0.4330
summer	0.6172	0.0	0.5512
classic	0.5487	0.0070	0.7025
party	0.0329	0.0	0.4152
*chic*	0.0	0.0	0.1235
*solid*	0.0	0.0	0.1810
*workout*	0.5424	0.2768	0.6215
*varsity*	0.0	0.1890	0.4329
print	0.8592	0.5124	0.8521
floral	0.6521	0.4540	0.6264
striped	0.6935	0.5829	0.7505
dot	0.3925	0.4150	0.4935
*linen*	0.0	0.0455	0.4163
*marled*	0.4	0.2722	0.6250
*leopard*	0.0	0.2966	0.3118
Average	0.3764	0.2600	0.5470

Italics: attributes with fewer data than others.

**Table 11:** Recall of 35 attributes using local multitask models with and without MCC.

Attribute	With MCC	Without MCC
lace	0.6996	0.6185
knit	0.7101	0.6606
denim	0.8035	0.8244
chiffon	0.6241	0.7538
dye	0.4688	0.3561
fur	0.7169	0.6654
metallic	0.2448	0.1573
sleeve	0.7235	0.6876
sleeveless	0.7093	0.6574
v-neck	0.5540	0.5702
collar	0.4377	0.3320
button	0.4193	0.4952
zip	0.5150	0.5173
bow	0.3140	0.3471
maxi	0.8560	0.8560
shirt	0.8480	0.8117
fit	0.6508	0.7127
bodycon	0.7436	0.7916
mini	0.5018	0.3593
midi	0.5383	0.4356
slim	0.5216	0.4330
summer	0.5856	0.5512
classic	0.6543	0.7025
party	0.4405	0.4152
chic	0.1235	0.1235
solid	0.1810	0.1810
workout	0.6045	0.6215
varsity	0.4878	0.4329
print	0.7578	0.8521
floral	0.7111	0.6264
striped	0.7542	0.7505
dot	0.5458	0.4935
linen	0.4785	0.4163
marled	0.5944	0.6250
leopard	0.4677	0.3118
Average	0.5711	0.5470

**Table 12:** Training, updating, and retrieving time table of the Clothes CFOR system with the DeepFashion dataset.

Phase	Option	Running time
Offline: learning phase (note: models can be trained individually to increase training speed)	Training object detection model	∼6 training hours for detection model
	Training object classification models	∼20 training hours for all 4 models in the system: 1 region identification model and 3 category classification models for Top, Bottom, and Body regions, respectively
	Training attribute learning models	∼22 training hours for all 5 grouped attribute multitask classification models in the system, including shape, part, style, texture, and fabric
	Updating system	∼2.5 training hours per model for all mentioned models in the training phase (1 region identification model, 3 category classification models, and 5 attribute multitask classification models)

Online: retrieval	Identifying object regions, categories, and attributes	2 to 3 seconds per sample
	Retrieving	1 to 10 milliseconds per sample (may delay by the run-time system)

**Table 13:** Full fine-grained attribute concept organization.

Groups of concepts		Fine-grained attribute concepts
Visual concepts	Color	red, purple, reddish orange, reddish purple, orange, purplish red, orange yellow, purplish pink, yellow pink, greenish yellow, yellowish pink, yellow green, brownish pink, yellowish green, brownish orange, green, reddish brown, bluish green, brown, greenish blue, yellowish brown, blue, olive brown, purplish blue, olive, violet, olive green
	Shape	a-line, a-line, ankle, asymmetric, asymmetrical, baja, bandage, beaded shift, bermuda, bib, big, bodycon, bodycon midi, box, box pleat, box-pleated, boxy, boxy crop, boxy knit, boxy lace, bustier, caged, cami, cami crop, cami maxi, capri, cargo, chiffon maxi, chiffon paneled, chiffon pleated, chiffon shift, chiffon-paneled, classic fit, classic skinny, combo, combo maxi, cover-up, cozy, crepe shift, crochet crop, crochet maxi, crochet-paneled, crop, cropped, cropped knit, cut, cutoff, cutout, cutout maxi, cutout sheath, denim pencil, denim shift, denim skater, distressed low-rise, distressed mid-rise, distressed skinny, drapey, embroidered fit, embroidered gauze peasant, embroidered maxi, embroidered peasant, embroidered shift, eyelet fit, faux leather mini, faux leather moto, faux leather paneled, faux leather pencil, faux leather skater, faux leather varsity, faux leather-paneled, faux-wrap, fit, fit flare, fit skinny, fitted, flare, flared, floral lace mini, floral lace sheath, floral lace skater, floral maxi, floral midi, floral mini, floral peasant, floral pleated, floral print skater, floral shift, floral skater, flounce maxi, flowy, fold-over, foldover, gaucho, gauze maxi, gauze peasant, graphic muscle, harem, high-low, high-rise, high-rise skinny, knee-length, knit longline, knit maxi, knit mini, knit pencil, knit skater, knit trapeze, kurt, lace maxi, lace midi, lace mini, lace pencil, lace sheath, lace shift, lace skater, leather mini, leather moto, leather pencil, leather skater, leather varsity, longline, longline shirt, low-rise, low-rise skinny, maxi, medium, mid rise, mid rise skinny, mid-rise, mid-rise skinny, midi, mini, moto, muscle, overlay sheath, oversized, peasant, pencil, pleated skater, polo, popover, print shift, print skater, print smock, print smocked, print tulip, puffer, pullover, raw, raw-cut, rise, rise skinny, rose skater, round, scuba skater, sheath, shift, shirt, skater, skinny, skinny stretch, skort, slim, slip, slouchy, smock, smocked, square, straight-leg, striped trapeze, swing, trapeze, trouser, tube, tulip, tunic, vertical, wide-leg, windbreaker, windowpane, wrap
	Texture	abstract, abstract chevron, abstract chevron print, abstract diamond, abstract floral, abstract floral print, abstract geo, abstract geo print, abstract paisley, abstract pattern, abstract print, abstract printed, abstract stripe, animal, animal print, bandana, bandana print, baroque, baroque print, bird, bird print, botanical, botanical print, boxy striped, breton, breton stripe, brushstroke, brushstroke print, butterfly, butterfly print, camo, camouflage, checked, checkered, cheetah, chevron, chevron print, chiffon floral, circle, clashist, classic striped, colorblock, colorblocked, crochet floral, daisy, daisy print, diamond, diamond print, ditsy, ditsy floral, ditsy floral print, dot, dots, dotted, embroidered floral, floral, floral flutter, floral paisley, floral pattern, floral print, floral textured, floral-embroidered, flower, foil, folk, folk print, geo, geo pattern, geo print, geo stripe, giraffe, giraffe print, graphic, grid, grid print, heart, heart print, heathered stripe, houndstooth, ikat, ikat print, kaleidoscope, kaleidoscope print, knit stripe, knit striped, leaf, leaf print, leave, leopard, leopard print, linen, linen-blend, mandala, mandala print, marble, marble print, marled, marled stripe, medallion, medallion print, mixed, mixed print, mixed stripe, mosaic, mosaic print, multi-stripe, nautical, nautical stripe, nautical striped, ombre, ornate, ornate paisley, ornate print, paint, paint splatter, painted, paisley, paisley print, palm, palm print, palm springs, palm tree, pattern, patterned, pinstripe, pinstriped, polka dot, pom-pom, print, print shirt, print woven, printed, ribbed stripe, ringer, rugby stripe, rugby striped, sophisticated, southwestern, southwestern-inspired, southwestern-patterned, southwestern-print, speckled, splatter, spotted, stripe, striped, stripes, structured, tonal, tribal, tribal-inspired, two-tone, varsity-striped, watercolor, zig, zigzag

Specific fashion concepts	Fabric	acid, acid wash, applique, bead, beaded, beaded chiffon, beaded sheer, bejeweled, bleach, bleached, bleached denim, brocade, burnout, cable, cable knit, cable-knit, canvas, chambray, chambray drawstring, chenille, chiffon, chiffon lace, chiffon layered, chiffon shirt, chino, chunky, chunky knit, classic cotton, classic denim, classic knit, classic woven, clean, clean wash, cloud, cloud wash, coated, corduroy, cotton, cotton drawstring, cotton knit, cotton-blend, crepe, crepe woven, crinkled, crochet, crochet embroidered, crochet knit, crochet lace, crochet mesh, crochet overlay, crocheted, crocheted lace, cuffed denim, cutout lace, damask, denim, denim drawstring, denim shirt, denim utility, dip-dye, dip-dyed, distressed, dye, elasticized, embellished, embroidered, embroidered gauze, embroidered lace, embroidered mesh, embroidered woven, embroidery, eyelash, eyelash knit, eyelash lace, eyelet, faded, fair, fair isle, faux, faux fur, faux leather, faux shearling, faux suede, feather, floral knit, floral lace, floral mesh, foulard, frayed, french, french terry, fur, fuzzy, fuzzy knit, gauze, gauzy, gem, georgette, gingham, glass, glitter, heathered, heathered knit, herringbone, jacquard, knit, knit lace, lace, lace layered, lace mesh, lace overlay, lace panel, lace paneled, lace pleated, lace print, lace sheer, lace-paneled, lacy, lattice, layered, leather, leather paneled, leather quilted, leather-paneled, led, loop, loose, loose-knit, mesh, mesh overlay, mesh panel, mesh paneled, mesh-paneled, metallic, mineral, mineral wash, neon, neoprene, nets, netted, nylon, oil, organza, origami, overlay, panel, paneled, patched, patchwork, perforated, pima, pintuck, pintuck pleated, pintucked, plaid, plaid shirt, pleat, pleated, pleated woven, pointelle, ponte, print satin, print scuba, purl, quilted, rhinestone, rib, rib-knit, ribbed, ribbed-knit, ripped, ruched, ruffle, ruffled, sateen, satin, scuba, seam, seamless, seersucker, semi-sheer, sequin, sequined, shaggy, shearling, sheer, sheer-paneled, shirred, shredded, sleek, slick, slub, slub-knit, sparkling, stone, stone washed, stones, stretch, stretch-knit, studded, suede, tapestry, tartan, terry, textured, textured woven, tie-dye, tiered, tile, tulle, tweed, twill, velvet, velveteen, waffle, wash, washed, woven
	Part	arrow collar, asymmetrical hem, back bow, back cutout, back knit, back lace, back striped, backless, batwing, beaded collar, bell, bell-sleeve, belted, belted chiffon, belted floral, belted floral print, belted lace, belted maxi, belted plaid, boat neck, bow, bow-back, bow-front, boxy pocket, braided, button, button-front, buttoned, cap-sleeve, chiffon surplice, cinched, classic crew, classic crew neck, classic pocket, classic v-neck, collar, collar lace, collared, collarless, collarless faux, colorblock pocket, contrast, contrast trim, contrast-trimmed, convertible, cowl, cowl neck, crew, crew neck, crisscross, crisscross-back, crochet fringe, crochet-trimmed, cross-back, crossback, cuffed, cuffed-sleeve, curved, curved hem, cutout-back, deep v-neck, deep-v, dolman, dolman sleeve, dolman-sleeve, dolphin, dolphin hem, double-breasted, drape-front, draped, draped open-front, draped shawl, draped surplice, drawstring, drop waist, drop-sleeve, drop-waist, dropped, elephant, elephant print, faux leather-trimmed, fitted v-neck, flat, flat front, flat-front, floral print strapless, floral print surplice, floral surplice, flounce, flounced, fluted, flutter, flutter sleeve, flutter-sleeve, fringe, fringed, gathered waistline, graphic racerback, heathered v-neck, hem, high-neck, high-slit, high-slit maxi, high-waist, high-waisted, hood, hooded, hooded maxi, hooded utility, illusion, illusion neckline, kangaroo, kangaroo pocket, keyhole, knit open, knit pocket, knit raglan, knit shawl, knit v-neck, knotted, lace peplum, lace sleeve, lace trim, lace-trim, lace-trimmed, lace-up, ladder-back, lapel, leather peplum, leather trimmed, leather-trimmed, long sleeve, long-sleeve, long-sleeved, m-slit, m-slit maxi, mesh racerback, mesh-trimmed, mock, mock neck, mock-neck, neck ribbed, neck skater, neck striped, neckline, notched collar, off-the-shoulder, one-button, one-shoulder, open-back, open-front, open-knit, open-shoulder, peplum, pin, pocket, print racerback, print strapless, print strappy, print surplice, print v-neck, racerback, raglan, raglan sleeve, ruffle trim, scallop, scalloped, scoop, scoop-neck, self-tie, shawl, shoulder, side slit, side-slit, single-button, sleeve, sleeveless, slit, split, split-back, split-neck, strap, strapless, strapless tribal, strappy, striped v-neck, surplice, suspender, t-back, tassel, tasseled, tie-back, tie-front, tie-neck, toggle, topstitched, trim, trimmed, tulip-back, turtle-neck, twist-front, twisted, two-button, v-back, v-cut, v-neck, vent, vented hem, y-back, zip, zip-front, zip-pocket, zip-up, zipped, zipper, zippered
	Style	americana, angeles, art, athletic, audrey, babe, babydoll, barbie, baseball, basic, basquiat, beach, beatles, bed, bella, bike, biker, blah, blurred, boho, bold, boyfriend, brooklyn, brooklyn nets, california, camera, candy, cardio, cat, chic, cities, city, civil, classic, coast, coffee, cute, dainty, daring, dark, darling, defyant, desert, destroyed, devil, doll, doodle, dream, dreamcatcher, dreamer, dynamite, eagle, edge, eiffel, elegant, enchanted, ethereal, everyday, fan, fancy, festive, field, fisherman, flawless, flirty, floyd, fox, france, free spirit, fresh, frida, galaxy, garden, garden party, genuine, girl, girls, grunge, guns, halen, heat, hepburn, heroes, inset, internet, island, isle, joie, kahlo, kid, killin, kiss, kitty, la, lady, lakers, laser, life, light, lightning, logo, lounge, love, lover, loyal, luxe, mandarin, map, marilyn, marilyn monroe, matelot, meow, miami, mickey, mickey mouse, mina, minnie, minnie mouse, mirrored, mob, mod, modernist, monroe, moon, morning, muse, new york, night, notorious, ny, nyc, oxford, pan, paradise, paris, party, performance, pineapple, pink, pizza, pj, please, popcorn, posh, power, quirky, rad, raga, rainbow, rebel, red, refined, regime, relaxed, retro, reverse, reversible, roll, rolling, rolling stones, roman, rose, roses, rugby, run, running, rustic, safari, sea, seaside, shark, shopping, shore, sky, smart, smile, snap, snoopy, soft, solid, spirit, spongebob, sporty, springs, standout, star, stars, studio, summer, sun, sunburst, sunflower, surfer, sweet, sweetheart, swim, swiss, taco, tasmanian, texas, thermal, tokyo, tower, track, training, tree, trench, triangle, tropical, trouble, tupac, utility, van, varsity, venice, voyager, wake, wave, weekend, west, wifey, wild, wildflower, woke, workout, yoga, yoke, york, youth, zeppelin

## Data Availability

The DeepFashion dataset used to support the findings of this study has been deposited in the Liu repository (https://drive.google.com/drive/folders/0B7EVK8r0v71pQ2FuZ0k0QnhBQnc). This dataset is under the MMLAB right, please follow their agreements and dowload instructions covered in: http://mmlab.ie.cuhk.edu.hk/projects/DeepFashion.html. The following datasets used to support the findings of this study or obtained from this study are currently under embargo, while the research findings are commercialized: structured cropped images in the DeepFashion dataset, imbalanced attribute dataset (IAD-35) filtered from the DeepFashion dataset, and extracted database for coarse-to-fine fashion object retrieval. Requests for data will be considered by the corresponding author, and data will be published at one month (to a maximum of 12 months) after publication of this article.

## Appendix

This section aims to fully express the organization (manually) of the fine-grained attribute concepts matched with each group of concepts (see Table 13 for more details). Note that the concepts and the organization of concepts can be added, edited, changed, or removed.
